# SLALOM, a flexible method for the identification and statistical analysis of overlapping continuous sequence elements in sequence- and time-series data

**DOI:** 10.1186/s12859-018-2020-x

**Published:** 2018-01-26

**Authors:** Roman Prytuliak, Friedhelm Pfeiffer, Bianca Hermine Habermann

**Affiliations:** 1 Computational Biology Group, Max Planck Institute of Biochemistry, Am Klopferspitz 18, 82152 Martinsried, Germany; 2 Computational Biology Group, Aix-Marseille University & CNRS, Developmental Biology Institute of Marseille (IBDM), UMR 7288, Parc Scientifique de Luminy, 163 Avenue de Luminy, 13009 Marseille, France

## Abstract

**Background:**

Protein or nucleic acid sequences contain a multitude of associated annotations representing continuous sequence elements (CSEs). Comparing these CSEs is needed, whenever we want to match identical annotations or integrate distinctive ones. Currently, there is no ready-to-use software available that provides comprehensive statistical readout for comparing two annotations of the same type with each other, which can be adapted to the application logic of the scientific question.

**Results:**

We have developed a method, SLALOM (for StatisticaL Analysis of Locus Overlap Method), to perform comparative analysis of sequence annotations in a highly flexible way. SLALOM implements six major operation modes and a number of additional options that can answer a variety of statistical questions about a pair of input annotations of a given sequence collection. We demonstrate the results of SLALOM on three different examples from biology and economics and compare our method to already existing software. We discuss the importance of carefully choosing the application logic to address specific scientific questions.

**Conclusion:**

SLALOM is a highly versatile, command-line based method for comparing annotations in a collection of sequences, with a statistical read-out for performance evaluation and benchmarking of predictors and gene annotation pipelines. Abstraction from sequence content even allows SLALOM to compare other kinds of positional data including, for example, data coming from time series.

**Electronic supplementary material:**

The online version of this article (10.1186/s12859-018-2020-x) contains supplementary material, which is available to authorized users.

## Background

Nearly all sequences have associated annotations, which describe continuous sequence elements (CSEs) with a specific function. In genomes, we have genes with their associated labels (coding regions, introns, exons, 5′ and 3’ UTRs, etc.), mapped and predicted binding sites for DNA-binding proteins (transcription factors, histone marks or other epigenetic features), or regions with a specific base composition or function (promoters, enhancers, CpG islands, repeat regions, etc.); in proteins, we find annotations like transmembrane regions, conserved domains, functional short linear motifs, or sites for protein modifications.

We are often faced with the problem of comparing such annotations. We need it whenever we want to compare the outputs from two distinct origins, such as genome annotations from two different resources or protein domains from two different predictors; or, when we want to integrate independent annotations with each other, such as transmembrane regions and motifs in proteins or genes and promoters in DNA. Annotations from two different origins may either be equally reliable, or one may be more reliable and thus be used for benchmarking. This is for instance the case, when we compare the results of a predictor to a golden standard of manually curated annotations. In this case, we want to compute performance measures. The measures are based on such counts as true positives (TP), false positives (FP), true negatives (TN) and false negatives (FN).

The terms ‘true positive’ and ‘false positive’ seem to be understood intuitively and thus these computations may seem to be a trivial task. However, considering different scenarios of overlap and duplication of annotated CSEs, their meanings may become quite ambiguous. Such ambiguity sources can be described by the following questions: (i) How should duplicated or overlapping CSEs within one annotation be resolved? (ii) What is a sufficiently large overlap between CSEs from two different annotations, so that they can be considered a match? (iii) How should length diversity among CSEs be treated? (iv) How should one account for the diversity in overall length of the sequences that have a CSE to be compared?

The answers to these questions depend on the particular problem under consideration. Let us first consider the way, how we can measure the overlap between two CSEs: one can either count a CSE as one single event, which we refer to as ‘CSE-wise’ or ‘site-wise’; alternatively, one can count each residue separately, so that the count depends on the length of the CSE. We refer to this as ‘symbol-wise’ or ‘residue-wise’. Depending on the type of application, either of the two models is typically used. For example, computing performance measures for predictors of protein secondary structure or solvent accessibility is usually done in a residue-wise manner, with CSE counts being rather irrelevant [1, 2]. On the other hand, in case of motif or domain predictions in proteins or gene annotations in genomes, it is more relevant to count CSEs as atomic units, without respect to their length. When comparing predicted conserved domains in proteins, Ghouila et al. [3] based their measures on the numbers of domains. The distance between two genomes is normally measured in numbers of rearrangements, regardless of their length [4]; in comparative genomics, it is more informative to compare genomes of different species in terms of gene counts rather than numbers of base pairs [5, 6]. In such situations, questions (i) and (ii) on the overlap and duplication of CSEs need to be carefully considered.

Song and Gu [7] generally outlined the approach for benchmarking de novo motif search algorithms: in brief, residue-wise measures complement the site-wise ones. For site-wise comparison of predicted motifs to a set of benchmark motifs, one must define a minimal overlap between the two motifs so that they can be considered a match. However, their proposed solution does not consider all the details (e.g., dealing with overlapping benchmark CSEs). Furthermore, their benchmarking software is not available as a standalone application.

Kalkatawi and colleagues [8] describe the problem of genome annotation comparison and provide a context-specific solution in the form of the software package BEACON. They suggest applying the length percentage threshold to classify a pair of compared genes as either matching or discrepant. By default, genes must overlap by at least 98% to be considered as a match. Their tool, BEACON, outputs the site-wise similarity score as the result. Other described solutions for comprehensive comparison of gene annotations are: the software package ‘GenoMatrix’ [9], annotation enrichment analysis [10], the GeneOverlap R package (part of Bioconductor [11]) developed in the lab of Li Shen (e.g. was used in [12]), diffReps – a specific solution for ChipSeq data [13], or bedtools [14], a standalone tool for a wide range of genomic analysis tasks. The most general existing solution is the IRanges R package (part of Bioconductor).

Questions (iii) and (iv) on the difference in length of the CSEs, as well as the full-length input sequences containing CSEs to be compared are potentially not so important, if one needs to compute performance measures for comparing just a pair of already finalized annotations. However, they become extremely important if one uses statistical measures as optimization criteria. For example, optimizing a motif predictor for a measure that includes residue-based recall may lead to a situation, where only the longest motifs are correctly recovered, while the shortest ones are being ignored. This is clearly not the desired behaviour. Optimizing for site-based measures, on the other hand, usually leads to prediction of overly extended motifs, which have an increased probability of covering the benchmark motifs just by chance.

Finally, one should consider, whether all sequences under consideration should be treated equally, as simple averaging of results across all sequences may not produce an adequate measure for the overall performance. Group-wise macro-averaging could for instance be desirable, if a dataset contains clusters of highly similar sequences (e.g. clusters of closely related homologs). In other cases, sequences may be grouped by a common feature, such as protein sequences belonging to the same complex, pathway, or the groups can represent regions with different properties in the same sequences – so-called class intervals [15]. To circumvent the grouping problem, one could select a single representative from each cluster or group. However, in this case, results could be biased due to the chosen representatives. Therefore, it is preferable to design the calculations such that all data are considered. As was pointed out by Baker et al. [16], estimation of statistics from grouped data does not raise principally new issues. Yet, various formulae need to be adjusted to reflect the nature of the data. A motif search is a good example, as each type of motif is normally present in more than one distinct sequence. In this case, sequences are grouped by containing the same type of motif.

We have developed a method, SLALOM (StatisticaL Analysis of Locus Overlap Method), for comparison of sequence annotations. By providing a set of different input options, SLALOM is tuneable to the relevant scientific question with respect to overlap and duplication of annotations, and provides the user with a number of statistical parameters relevant for performance measures. We have tested SLALOM on different annotation comparison scenarios, which we present in this manuscript. Moreover, we have written SLALOM in such a way that it cannot only be applied to positional data representing sequence annotations, but can also be used for comparing time-series data.

## Results

### Results overview

**Fig. 1:**
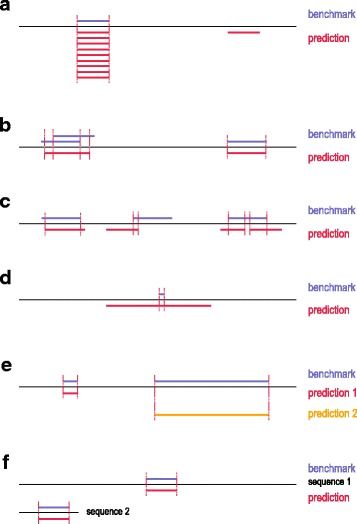
Overview of possible ambiguities, when comparing two annotations of CSEs (benchmark and predicted CSEs). Black lines depict query sequences, blue lines indicate benchmark CSEs, red and orange lines represent predicted CSEs. **a** Multiple true positive sites (left) and a single false positiv site (right). **b** A true positive matches to multiple, overlapping benchmark sites (left) or to a single benchmark site (right). **c** The overlap between a predicted site and a benchmark site may be large (left), minimal (center) or one predicted site may patch multiple benchmark sites (right). **d** An excessively large predicted site overlaps with a short benchmark site. **e** Two predictors have one true positive and one false negative; the matching benchmark site may be short (left) or long (right). **f** A predictor finds a benchmark site in either a long sequence (top) or a short sequence (bottom). For more details, see Main Text

When two annotations of CSEs are compared, different scenarios of overlap and duplication may lead to quite some ambiguity during evaluation. Several scenarios are illustrated in Fig. 1. We start with a description of the details of these scenarios, which is the motivation for all other results that we have obtained.

We have designed and implemented comprehensive overlap resolving and matching principles to cope with the ambiguity during evaluation. Each CSE has its length. Depending on the kind of analysis, it can be viewed as a single event, independent of its length, or as a multitude of events proportional to its length. In some analyses, it is only relevant, if there is a CSE at a given position or not (binary event), while in others, the exact counts are important (e.g., so-called deepness in next-generation sequencing (NGS)). Finally, a pair of CSEs may come from two annotation origins with equal confidence; or one of them might be more reliable (e.g., be considered the golden standard or benchmark). To address these different analysis types, we have implemented three count modes, which can each be combined with two comparison modes, resulting in a total of six operation modes (Table 1). Both, the count and the comparison modes are mutually exclusive. Full details of these operation modes are presented below.

**Table 1:** Operation modes of SLALOM. Each input is parsed twice, so that each annotation is at one point the query and the subject, respectively

Mode	Description
**Count modes (mutually exclusive, collectively exhaustive)**	
Symbol-resolved	While calculating symbol-wise statistics, classify symbols to either present or absent in the query annotation. Calculate site-wise statistics according to the overlap logic. This is the default mode.
Gross	While calculating symbol-wise statistics, count each symbol gross, i.e., as many times as it occurs in all sites from the query annotation. Calculate site-wise statistics according to the overlap logic.
Enrichment	While calculating symbol-wise statistics, classify symbols to either enriched or non-enriched (including completely absent) in the query annotation based on the user-provided threshold on the number of occurrences. Do not calculate site-wise statistics.
**Comparison modes (mutually exclusive, collectively exhaustive)**	
Equal	Treat the two input annotations as equal. Calculate only symmetric (not influenced by swapping) performance measures.
Benchmarking	Treat the first input annotation as the benchmark; treat the second one as a prediction. Calculate both symmetric and non-symmetric performance measures.

We demonstrate the applicability of our tool in three case studies. The first case study deals with the annotation of proteins. It analyses some details of the performance of our previously published method HH-MOTiF, a de novo motif predictor [17]. We also compare the functionality of SLALOM to other available tools by addressing specific questions within this case study. In the second case study, we compare the annotations of two prokaryotic (archaeal) genomes with respect to calling of protein-coding genes. The third case study illustrates the applicability of the tool to data from a time series. It is an analysis of economic data, showing that our statistical analysis tool is not restricted to biological data.

### Identified sources of ambiguity when comparing CSEs from two annotation origins

By carefully analysing examples of annotation comparisons available in literature, as well as in published software solutions, we identified four distinct sources of ambiguity:

1. Overlaps and duplications between CSEs in the same annotation
2. Criteria for matching of CSEs from different annotations
3. Length diversity among distinct CSEs
4. Length diversity among the annotated sequences

Thus, the four corresponding questions, which refer to sources of ambiguity, should be clarified before calculating any performance measures.

1. *How should duplicated and overlapping CSEs in the same annotation be resolved?* CSE overlaps and duplications are integral to some problems, e.g., exon annotations. However, they may be unwanted artefacts, as is the case for motif predictions. Let us assume that there is only one benchmark motif, which is correctly recovered by the predictor; however, if the predictor outputs it nine times (as duplicates) in addition to one distinct false positive (see Fig. 1a), is the precision of the predictor 90% (counting each duplicate separately) or 50% (consolidating duplicates)? Or maybe 100%, as one could discard the second predicted motif as non-significant based on the duplicate count? Moreover, how should one resolve overlaps in the benchmark annotation itself (see Fig. 1b): should one merge the overlapping sites or treat them as distinct sites?
2. *To which extend must the CSEs of two annotations overlap to be considered a match?* This question addresses the problem of finding unequivocal matches between the annotated CSEs. In case of motif prediction, it is very convenient to speak about certain benchmark motifs being either ‘correctly recovered’ or ‘missed’ (see for instance [18]). It is a clear-cut situation, when a benchmark motif almost perfectly corresponds to a predicted one (Fig. 1c, left). Yet, can one still count a motif as ‘correctly recovered’, if it overlaps with a predicted motif only to a small extent (Fig. 1c, centre)? Or if it is ‘patched’ by several different predicted motifs (Fig. 1c, right)? If not, what threshold should be applied? A typical sub-problem is dealing with predictors that output very long motifs to hit the benchmark motifs just by chance (Fig. 1d).
3. *How should length diversity among annotated CSEs be treated?* This question deals with the problem of considering a CSE as an atomic unit or as a collection of the separate symbols it consists of. Let us assume that two CSEs to be compared have very different lengths. Does a prediction, which recovers only the shorter CSE perform equally well as a prediction, which recovers only the longer one (Fig. 1e)?
4. *How should length diversity among the compared full-length sequences be treated?* This question addresses the statistical significance of a prediction with respect to the sequence space it resides in: returning to the problem of de novo motif prediction, should a correct prediction of a motif in a significantly longer sequence be considered statistically more significant than another correct prediction of the same motif in a much shorter sequence (Fig. 1f)?

We do not pretend to provide an exhaustive list here. Other potential sources of ambiguity can be identified, when comparing two annotations of CSEs. In this study, we focus our attention on those that have potentially the largest impact with respect to biological data. However, SLALOM can to some extend also handle other ambiguity sources, such as missing values or group size inequality. For full details on the functionality of SLALOM see Methods, Additional file 1: Table S1, and the user manual in Additional file 2 (also downloadable from GitHub).

### Implemented operation modes and their applicability

**Fig. 2:**
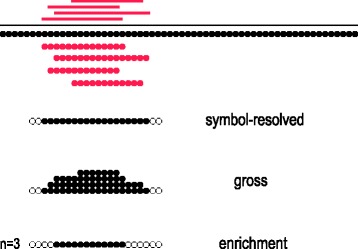
Schematic representation of differences between the three count modes. The grey line represents a query sequence; red lines show overlapping CSEs in this sequence; circles illustrate distinct symbols (residues, base pairs, time points, etc.) the sequence consists of. The symbol-resolved mode counts presence of at least one symbol in a position; the gross mode counts how often each symbol position occurs; the enrichment mode is similar to the symbol-resolved mode but counts presence only if there are at least ***n*** symbols in a position

The ambiguity source 1 – overlaps of CSEs within one annotation – can be addressed in two ways according to the user’s choice. The first approach consists in resolving the overlaps through either merging overlapping CSEs or discarding the redundant ones. It is invoked through changing the default of the options ‘-a1r/--anno1file_resolve’‘-a1r/--anno1file_resolve’ and ‘-a2r/--anno2file_resolve’ (see Additional file 1: Table S1). The second approach consists in counting the number of CSEs traversing each symbol. This counting may be done in three modes (see Table 1 and Fig. 2): (1) by ‘presence’ (either traversed or not, as if merging were performed). We refer to this as symbol-resolved mode; (2) in ‘gross’ mode (each symbol is counted as many times as it is traversed); or (3) by ‘threshold’ (the symbol is counted as present if it is traversed by at least some defined minimal number of CSEs). We call this enrichment mode. Note that real explicit merging and counting for presence, although producing identical symbol-wise results, will lead to generally different site-wise metrics. For the list of all metrics available for calculation in each mode, see Table 2.

**Table 2:** Performance measures availability in different modes. For the formulae of the metrics, see Module 4 and Module 6 of Methods

Measure	Symmetric	Operation mode					
		Symbol-resolved		Gross		Enrichment	
		Equal	Benchmarking	Equal	Benchmarking	Equal	Benchmarking
TPR	no	–	+	–	+	–	+
PPV	no	–	+	–	+	–	+
SPC	no	–	+	–	+	–	+
NPV	no	–	+	–	+	–	+
Informedness	no	–	+	–	+	–	+
Markedness	no	–	+	–	+	–	+
PC	no	–	+	–	+	–	+
ACC	yes	+	+	–	–	+	+
MCC	yes	+	+	–	–	+	+
F1	yes	+	+	+	+	+	+
EAC	yes	–	–	–	–	+	+
Site TPR	no	–	+	–	+	–	–
Site PPV	no	–	+	–	+	–	–
Site PC	no	–	+	–	+	–	–
Site F1	yes	+	+	+	+	–	–
Site PCV	yes	+	+	+	+	–	–

The ambiguity source 2 – matching criteria for CSEs from two different annotation origins – is addressed by allowing users to set the matching criteria: minimal number of symbols with the option ‘-Os/--overlap_symbols’ and minimal overlapping part (fraction) with the option ‘-Op/--overlap_part’. This standard functionality is also available in other published tools. The possibility to unambiguously define, to which of the two CSEs these criteria apply (the option ‘-Oa/--overlap_apply’), is, however, a unique feature of SLALOM. It also offers the possibility to define the desirable order of the CSE start positions or which type of events should begin earlier in a time series. In this case, two CSEs only match, if the CSE from the second annotation begins before, after, or at the start position of the corresponding CSE from the first annotation (option ‘-On/--overlap_nature’). Moreover, the shift options (‘-a1bs/--anno1file_begin_shift’, ‘-a1es/--anno1file_end_shift’, ‘-a2bs/--anno2file_begin_shift’, ‘-a2es/--anno2file_end_shift’) allow matching of CSEs that are not overlapping but are merely close to each other, as well as for compensating for possible annotation skews. Such functionality is especially useful for tasks like gene-promoter matching or gene name mapping between two different genome annotations based on their relative position in the genome.

The ambiguity source 3 – CSE length diversity – is addressed through computing both, residue-wise and site-wise measures. The latter will show underperformance in comparison to the former, if the predictor selectively prefers longer CSEs.

**Fig. 3:**
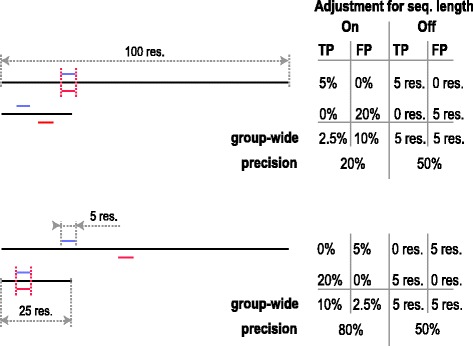
Schematic example of evaluating a predictor with and without adjusting for sequence length. Black lines illustrate two query sequences (100 and 25 residues long). Two benchmark CSEs (both 5 residues long) are drawn as short blue lines; two predicted CSEs (also 5 residues long) are shown as short red lines. In the upper panel, the prediction worked correctly in the longer sequence but not in the shorter, and vice versa in the lower panel. With sequence length adjustment turned on, the actual residue counts are divided through the sequence length before proceeding to averaging and calculating performance measures. Otherwise, residue counts are summed up. The precision is computed as TP/(TP + FP)

The ambiguity source 4 – sequence length diversity – is addressed through the choice between turning the adjustment for the sequence length on (with the option ‘-A/--adjust_for_seqlen’) or off (the default). The former will convert the symbol counts (TP, FP, etc.) into percentages (or shares) of the sequence length for each sequence individually before averaging them group-wide or dataset-wide. The latter will sum up the counts group-wide before converting them into shares. With adjustment for sequence length turned on, the relative number of symbols is considered, rather than their absolute counts. As a result, for CSEs of equal length, the performance in shorter sequences outweighs the performance in longer sequences, if the adjustment is turned on. A schematic example of the impact of sequence length adjustment on the resulting metrics is shown on Fig. 3. Note that although the adjustment for sequence length can be viewed as macro averaging when calculating the shares of TP, FP, etc. at the group level, we do not use the term ‘macro averaging’ in this context in SLALOM, to avoid confusion with averaging of performance measures, which has a different impact on results. For the performance measures, we implement three averaging approaches: sequence-wide (macro-macro), group-wide (micro-macro; the default) and dataset-wide (micro-micro), which can be chosen with the option ‘-a/--averaging’.

The detailed description of the options is provided in Additional file 1: Table S1.

### Case study 1: Protein motif prediction as exemplified by application of the de novo predictor HH-MOTiF

#### Glossary

*Sequences*: protein sequences containing experimentally verified motifs.

*Groups*: separate motif classes (ELM [19] classes).

*Benchmark annotation*: ELM annotation of experimentally verified motifs.

*Predictor annotation*: output of a computational motif predictor (HH-MOTiF).

A previous version of SLALOM was used in an earlier publication [17] to assess the performance of different methods for de novo motif prediction in protein sequences, and to compare them between each other. In brief, we used experimentally validated motifs stored in the ELM database to develop, optimize and test the HH-MOTiF algorithm. Our goal was to make our predictions match benchmark motifs annotated in ELM as closely as possible. The difficulties in scoring predicted short motifs in proteins are given by the following factors corresponding to the ambiguity sources described in the previous subsection:

1. The motif instances predicted by HH-MOTiF are often overlapping or duplicated. Benchmark motifs annotated in ELM are also sometimes overlapping, even within the same motif class (e.g., in the ELM class LIG_SH3_3). It is not initially obvious, if one should merge the instances or treat them separately.
2. Sometimes benchmark and predicted motifs overlap only to a small extent. It is not clear, if one should still consider them as matches or simply ignore such overlaps.
3. The length of benchmark motifs, as well as the number of motif instances per class broadly varies. This may skew the final score in favour of predicting longer and/or more abundant motifs.
4. The length of proteins is highly diverse, also within the same motif class. This means that in different sequences, ratios between positive and negative residues may be more than 10 times different. As such ratios constitute the formulae of performance measures, two predictions of the same motif with equal absolute numbers of true positive and false positive instances will show quite different scores, depending on the distribution of the instances among the proteins. Therefore, one has to decide whether to focus on the motif count or the motif residue count (see Fig. 3).

We chose to calculate different measures for estimating the accuracy of selected de novo motif predictors to avoid biasing results in favour of one or the other method. We calculated residue-wise recall, residue-wise specificity, site-wise recall, site-wise precision, and site-wise performance coefficient (PC) in the symbol-resolved mode. Residue-wise precision was calculated in the gross mode. For details on calculations, see Methods.

Let us first consider calculating residue-wise performance metrics. The choice of how to treat overlaps in predicted motifs with benchmark motifs and how to calculate averages for performance metrics may seem trivial at the beginning. However, the impact of these choices may be as large as 2-fold. For example, precision (PPV) is very sensitive to the way of treating *nan*s during averaging, while the false positive rate (FPR; FPR = 1-SPC, with SPC being specificity) changes upon switching between the symbol-resolved and gross modes (see Table 3). Consequently, the performance values vary with the chosen application logic. The choice of the operation mode should be based on the question the researcher wants to answer. In case of motif predictors, we wanted the precision to answer the following question: “What is the probability that a given predicted motif is real?”. With this question, it is not important, if the given motif overlaps with others from the same annotation, and therefore we have chosen the gross mode to calculate PPV. In this application logic, a duplicated false positive prediction will decrease the precision. On the other hand, while calculating residue-wise recall (TPR), SPC and FPR, we wanted to answer the question: “What share of motif/non-motif residues are predicted as positive/negative?”. This is not influenced by duplications of some residues. Thus, we calculated TPR, SPC and FPR in the symbol-resolved mode. Moreover, we did all the calculations without adjusting for sequence length. This prevents generally easier cases of short sequences from outweighing the harder ones: we observed that it is harder to find short motifs in long proteins than in short ones. Without sequence length adjustment, the result depends only on the number of true and false positives in the group, regardless of their distribution between distinct sequences. With sequence length adjustment turned on, the sequence-based distribution of motifs would impact the performance, which we consider as an undesired effect in this situation. In our schematic example in Fig. 3, the precision is identical (50%) when the adjustment for sequence length is turned off but fluctuates between 20% and 80% when it is turned on. Finally, we did not treat *nan* values as zeros while calculating PPV. These arise when a predictor returns no results for a given motif class. We reasoned that it is better for a tool to predict no motifs at all than only false positives. If one treats *nan*s as zeros, these two cases become non-distinguishable. Taken together, our precision value answers the question “What is the probability that a given de novo predicted motif corresponds to a benchmark motif, independent of other predicted motifs?”. In our opinion, this is the most likely question an average user of such a tool will want to address.

**Table 3:** Dependence of the core performance measures of HH-MOTiF on the approach. Generation of this table is based on the option set A1 and its variants, as specified in Additional file 1

Operating count mode	Adjustment for sequence length	Treat *nan*s as zeros	Symbol-wise		
			TPR	PPV	FPR
Gross	no	no	0.211	0.420	0.011
		yes	0.211	0.213	0.011
	yes	no	0.216	0.429	0.014
		yes	0.216	0.217	0.014
Symbol-resolved	no	no	0.210	0.358	0.007
		yes	0.210	0.181	0.007
	yes	no	0.215	0.367	0.009
		yes	0.215	0.185	0.009

**Fig. 4:**
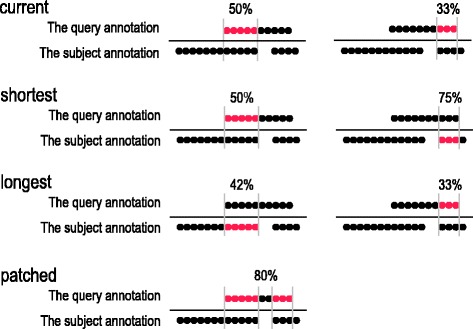
Schematic example illustrating principles of CSE matching criteria. The length of the CSE in the annotation being currently considered (the query) is 10 symbols/residues. It partially overlaps with two CSEs - the match candidates - in the subject annotation: with a 12-symbol long CSE by 5 symbols; and a 4-symbol long CSE by 3 symbols. In all four scenarios, both match candidates are evaluated to determine, if the current CSE has a match or not. In the first three scenarios (*current*, *longest*, *shortest*), they are tested separately, while they are treated cumulatively, if *patched* is selected. If at least one test succeeds, the current CSE has a match, otherwise - not. For example, if the user sets a length threshold of 60%, the current CSE has no match when selecting *current* or *longest,* but has a match if *shortest* or *patched* is chosen

Calculating site-wise TPR and PPV may be even more relevant, as it is more interesting to evaluate entire motifs than individual residues. However, the calculation of site-wise metrics is more ambiguous, as one needs to set the minimal overlap criteria for assigning a match between a predicted and a benchmark motif. There are many opinions on how well a benchmark-prediction motif pair should overlap to be counted as a match, or if a match must be reciprocal. In the HH-MOTiF paper, we chose the loosest definition, stating a reciprocal match, if the pair overlapped by at least one residue. With the newly implemented options in SLALOM, we can conduct more in-depth investigation of the site-wise performance. The options include not only the minimal required number ***N*** of residues and the minimal percentage ***P*** of a matching CSE (motif), which we refer to as ‘the criteria’, but also how they should be applied to the query and subject CSE (motif) to be compared (Fig. 4). This is important to consider, when the motifs are of different length. There are four possible options available (note that the input is considered twice, so that each annotation becomes the query and the subject annotation at one time):

- *current*: apply the criteria to the motif in the current annotation being considered – the query annotation. Consider only one motif from the other – the subject – annotation at a time.
- *shortest*: consider one motif at a time from the subject annotation and apply the criteria to the shorter of two motifs in the compared pair.
- *longest*: similarly to the *shortest*, except apply the criteria to the longer of two motifs in the compared pair.
- *patched:* apply the criteria to the motif in the currently considered annotation. Consider all motifs from the subject annotation cumulatively, allowing single query motifs to be ‘patched’ by several motifs from the subject annotation. The benchmark motif in Fig. 1c (right) may be considered as not recalled if the *current* is chosen but recalled if the *patched* is chosen.

SLALOM allows the user to define the matching principles according to his or her preference to obtain relevant data on the site-wise performance of a predictor. The dependence of performance measures of HH-MOTiF on ***N***, ***P*** and the chosen application logic is shown in Table 4.

**Table 4:** Site-wise performance of HH-MOTiF depending on the benchmark-prediction overlap logic. Generation of this table is based on the option set A1 and its variants, as specified in Additional file 1

***N***	***P***	Applying to	TPR	PPV
1	0	current^a^	0.236	0.564
	25	current	0.233	0.557
		shortest	0.233	0.558
		longest	0.231	0.557
		patched	0.233	0.557
	50	current	0.226	0.504
		shortest	0.229	0.552
		longest	0.214	0.484
		patched	0.226	0.508
	75	current	0.194	0.303
		shortest	0.220	0.516
		longest	0.136	0.226
		patched	0.194	0.304
3	0	current^b^	0.229	0.545
		patched	0.229	0.545
5	0	current^b^	0.178	0.397
		patched	0.179	0.399

^a^all four options are equal for these ***N*** (minimal required number of residues) and ***P*** (minimal percentage of a matching CSE)^b^current, shortest, and longest are equal for these ***N*** and ***P***

Data we received on the dependence of performance on the chosen application logic is itself informative about the properties of the input data. For instance, on the basis of Tables 3 and 4, we could make some fundamental observations: first, there are not many overlaps and duplications in the benchmark dataset in contrast to the predicted dataset. This is based on the observation that the residue-wise PPV is influenced to a much greater extent than the TPR by switching between gross and symbol-resolved modes. Closer inspection of the input data confirms this hypothesis (see input files in Case Study 1 in Additional file 1). Second, the predictor performs better on shorter proteins. This is based on the observation that the performance is slightly lower with adjustment for sequence length turned on. This hypothesis is consistent with our earlier observation that predictions in shorter proteins are easier, although the effect is very small for HH-MOTiF. Third, the predictor returns no results for about half of the groups: there is an about 2-fold impact on the symbol-wise PPV by treating *nan*s as zeros. Indeed, HH-MOTiF returned no results for 87 out of 176 tested motifs (see Additional file 1: Table S3 of the HH-MOTiF paper). Fourth, the predictor has generally no problems with correct positioning of the motifs (i.e., avoiding situations depicted in Fig. 1c, centre). This is based on the observation that both, site-wise TPR and PPV drop less than 10%, when requiring at least 75% of the shortest motif in the benchmark-prediction pair to overlap. Finally, the predictor often fails to reproduce precisely the annotated length, predicting either too short or too long motifs (Fig. 1d). This hypothesis is based on the significant drop of site-wise PPV upon requiring at least 75% of the longest motif in the benchmark-prediction pair to overlap.

For details and files, see also Case Study 1 in Additional file 1.

### Case study 2: Comparison of ORF calling from two independent genome annotations

#### Glossary

*Sequence*: chromosome sequence of the archaeon *Natronomonas pharaonis.*

*Groups*: reading frame categories (which includes strand selection).

*First annotation*: Genome annotated by manual curation in our previous works [20, 21] as submitted to GenBank [22].

*Second annotation*: Genome annotated by RefSeq [23].

Rapid expansion of sequencing capacities allowed for the rise of big data genomics. As of June 2017, around 25,000 organisms were fully sequenced (data from NCBI [24]). However, to extract useful biological information, genomic sequences have to be annotated. The process of annotation consists in marking positions of functional elements within genome sequences. The functional elements of the highest interest are genes – the sequence stretches that encode proteins and other biologically active compounds. In our case study, we looked at gene prediction, which is a key step in genome annotation. Because three genome residues encode one protein residue without punctuation, there are six potential reading frames, three in each direction, in every genome region. In eukaryotic organisms, genes can overlap and/or consist of several non-consecutive parts (exons). Even the so well studied genome of fruit fly (curated by FlyBase) is still subject to frequent revisions [25]. In addition, assigned gene identifiers (accessions) vary between different annotating bodies or even between different releases by the same body (e.g., FlyBase). Therefore, biological researchers often need to consider the discrepancies, if several versions of the genome of their interest are available. Here we demonstrate that the presented method can deal with both, positional and naming discrepancies of annotated genomic features, given that the annotations are made for the same release of the genome. For reasons of simplicity we have used a prokaryotic genome, more specifically the one from the archaeon *Natronomonas pharaonis*.

SLALOM provides two useful results: overall statistics of the annotation similarity, which is in this case usually close to but not exactly 100%; and the list of CSE (gene) matches between the two annotations. The latter can be also used to map the names on the basis of positional similarity. Alternatively, the list can be limited only to unmatched or discrepant genes to focus on the differences.

In the provided example, we mapped 2694 protein-coding genes from the GenBank annotations to 2608 genes from the RefSeq annotation of the genome sequence of *Natromonas pharaonis*. This genome is quite dense, which is typical for microorganisms, as 89.79% of all base pairs are part of an annotated gene in both annotations. We ran the comparison in the symbol-resolved and the gross modes. Genes from compared annotations were matched, if they overlapped by at least 50% of the length of the gene under investigation (*current* gene).

According to the expectations, the genomes are highly similar (F1 and ACC exceeding 98% in all modes). As part of the output, we obtained a map of gene identifiers between the two annotation origins. We encountered a few ambiguities, where SLALOM’s functionality came in helpful. As it can be seen from Table 5, some overlapping genes within the same genome were seen in both annotations (otherwise there would be no difference between total gene length in the symbol-resolved and gross modes, which treat overlaps in a different manner). However, the overlaps can mostly be explained by differences in reading frames. Exceptions are just 4 base pairs in the RefSeq genome, which arise from the overlap with a pseudo gene.

**Table 5:** Statistics on two genomes comparisons

Measure	Without frame separation		6 distinct reading frames	
	Symbol-resolved(option set B1)	Gross (option set B2)	Symbol-resolved (option set B3)	Gross (option set B4)
GenBank total gene length	2,360,518	2,367,915	2,367,915	2,367,915
RefSeq total gene length	2,336,204	2,339,349	2,339,345	2,339,349
Symbol-wise ACC	0.9861	–	0.9967	–
Symbol-wise F1	0.9923	0.9923	0.9892	0.9892
Site-wise F1	0.9779	0.9779	0.9751	0.9751

The rise in ACC upon dividing the genes into 6 classes based upon the reading frame is attributed to a form of the false positive paradox. While the total sequence length remains unchanged, the number of annotated residues is getting less. As the false positive and false negative counts are more or less proportional to the overall positive count, the accuracy rises accordingly. The F1 score, on the other hand, is not subjected to the false positive paradox and shows a decrease upon the division into classes. This decrease is caused by the fact that some matches between different classes are not counted any longer. Furthermore, the equality of F1 scores between symbol-resolved and gross modes is not guaranteed; the fact that they are equal up to the 4th point means that gene overlaps are generally – or perhaps completely – the same in the two annotations.

Examples files and further details can be found in Case study 2 in Additional file 1.

### Case study 3: Analysis of a time series as exemplified by analysis of economical data

A potential application of SLALOM is to analyse data from epidemiological studies as consecutive series of events (e.g., decreases in temperature as putative causes and spikes in disease or mortality rates as putative consequences [26]), as well as from appearing and disappearing of symptoms in the course of a disease progression (or psychological condition, as, for example, in [27]) in a cohort of patients. The options ‘shifting’ start and stop time point (see the option ‘-a1bs/--anno1file_begin_shift’ in Additional file 1: Table S1) allow detecting events (CSEs) related by assumed causality even with significant time lags. However, as we did not have a large enough clinical dataset at our disposal, we show the possibilities of the proposed method on non-biological time series data, demonstrating the general applicability of our tool. In brief, we looked for possible causality relations between economical news releases and movements in currency exchange rates. News data were extracted from the event database (FXStreet.com). For exchange rates (EUR to USD) we used open, high, low and close (OHLC) values for 1-min intervals throughout the calendar year (downloaded from HistData.com). From the OHLC data we computed start and finish time points of the trends (time intervals of rapid directional price movements) and inspected, if such trends would correlate with the appearance of economical news. We demonstrate that there is no evidence that news releases precede strong price movements (which can be clearly seen from Additional file 1: Table S3). Full details are provided in Case study 3 in Additional file 1.

### Comparison to other CSE analysis methods

Software tools, which are similar to SLALOM, assess overlaps between annotation features and are freely available, include BEACON, GeneOverlap (part of R BioConductor), and diffReps. Albeit performing similar calculations to our methods, GeneOverlap and diffReps evaluate the resulting statistics from a different angle and have different purposes than SLALOM. Both calculate *p*-values to assess significance of isolated associations in gene annotations and/or ChIP-Seq peaks. Moreover, both methods have a much narrower scope of application. Therefore, we find them not generally comparable with our method. However, BioConductor contains other functions, which can replicate the functionalities of SLALOM to a significant degree. Thus we compared our method with BioConductor. Especially close is the IRanges package. It should be noted that working with BioConductor requires actual writing of R scripts, including, among others, handling of data import and export, and thus also basic knowledge of R as a programming language. In contrast, our method operates as a standalone Python application and can do complete analyses with only one command line. In addition, we compared SLALOM to BEACON and bedtools. As one can see from our short summary on functionality (see Table 6), BioConductor is the most comprehensive package for annotation comparisons. However, it is quite tricky to actually apply it for some problems without writing a substantial amount of R code, although it contains the library IRanges for generic types of data. Among others, evaluation of protein motif predictors and time series correlation analysis, which are illustrated by our case studies are not readily doable with BioConductor.

**Table 6:** Overview of the functionality of the discussed tools for comprehensive annotation comparisons

		SLALOM (v2.1.4)	Bioconductor (version 10/2017)	bedtools (v2.25.0)	BEACON (as in [8])
Overlap sufficiency criteria to match	Symbol count	yes	yes	no	no
	Length share	yes	no	yes	yes
	Order	yes	yes	no	no
	Enrichment	yes	yes	yes	no
Match by proximity without overlap		yes	yes	yes	no
Application principle of the overlap criteria	Shortest	yes	no	no	yes
	Longest	yes	no	no	no
	Current	yes	yes	yes	no
	Patched	yes	yes	no	no
Resolving overlaps within single annotation	Merge	yes	yes	yes	no
	Disjoin	no	yes	no	no
	Leave one	yes	no	no	no
	By enrichment	yes	no	yes	no
Residue-wise statistics	Symbol-resolved	yes	yes	no	no
	Gross	yes	no	no	no
Site-wise statistics		yes	yes	yes	yes
Performance measures		yes	no	no	no
Jaccard statistics		no	yes	yes	no
Sequence grouping		yes	yes	yes	yes
Combining annotations		yes	yes	yes	no
Time series processing		yes	no	no	no

We have developed a BioConductor-dependent R script (available as Additional file 3) for answering the three following questions: (1) What share of residues in a motif-containing sequence does belong to motifs of a specific type? (2) What share of motif instances described in the older version of the ELM database has exact matches in a newer version? (3) What share of motif instances described in the older version of the ELM database are at least to 50% covered by a distinct motif instance of the same ELM class in the newer version?

One single command choosing specific options (D1, Additional file 1) addresses questions 1 and 2 using SLALOM: on average, 1.23% for the older version and 1.18% for the newer version of the length of motif-containing protein sequences contains a motif of a specific type; 92.55% of the motif instances in the older version of ELM have exact matches in the newer one. Questions 3 can be addressed by choosing a different set of command line options (D2, Additional file 1): 93.49% of the motif instances in the older version are at least to 50% covered by a distinct motif instance of the same ELM class in the newer version of ELM.

We could execute most of the comparisons by using BioConductor. However, there are two major implementation challenges in R, which we discuss briefly in the next paragraphs.

There is no easy way in BioConductor to classify ranges on the basis of overlap share with a potentially matching range from another GRanges object. Only classification on the basis of residue counts can be specified in the built-in functions (e.g., *countOverlaps*), by using the keywords ‘maxgap’ and ‘minoverlap’ with integer numbers. The *intersect* function cannot be used for this task, as it does not distinguish between matches for repeated, perhaps even overlapping, motifs in the same sequence. The *pintersect* function also cannot be used, as an equal number of motifs in the old and new version of the ELM database is not guaranteed. As a result, the user would have to loop over all the motif instances individually to answer question 3, which goes beyond the applicability of IRanges and GRanges objects. Therefore, we consider this question unanswerable in a simple way with BioConductor.

Second, large databases require additional handling. The sequence length database is quite long (more than 90 million records), although only a few thousand records are relevant for the comparison discussed here. There is no straightforward way to do the required filtering while reading in the sequence length file in R. Here, we first read the file into a data frame and then filter it for better performance of consecutive operations. If the file was even bigger, additional memory limitations could arise depending on the computational setup available to users. In this case, the BioConductor user needs to additionally code a line-by-line reading of the file. This is already implemented in SLALOM, so that the user can work with original databases that are larger-than-memory.

bedtools addresses the core of all three questions with the sub-tool *coverage*. It is fast and optimized for handling of large files. It also has flexible options for specifying overlap shares, although for some reason, the options for determining overlaps on the basis of residue counts are missing. In this sense, the functionality of bedtools is complementary to that of BioConductor; SLALOM, on the other hand, provides both possibilities out-of-the-box.

Despite the good handling of the core overlap counting problem, there are still two major challenges for the user, who wants to answer the given questions with bedtools. First, it accepts only specified formats; therefore, the user will have to convert the ELM and Uniprot [28] TSV files into one of the supported formats, e.g., BED. Second, bedtools has a limited arsenal of summary statistics: for example, it does not calculate averages and shares; therefore, the user will have to post-process the output to actually obtain the required numbers.

SLALOM has even more advantages, if one needs to compare a predictor against a ‘golden-standard’ database rather than comparing two datasets to each other. In this case, one needs not only to handle the overlaps, but also to calculate benchmark statistics, such as accuracy or F1 values. Neither of these calculations in the complexity we describe them here are easily doable with BioConductor or available for bedtools.

In general, one can observe that BioConductor and bedtools represent powerful solutions for a broad scope of genomic comparisons and analyses. In this area, their functionality goes far beyond that of our method. However, they are not designed to answer a broad range of statistical questions about the underlying data. For instance, these tools are not designed to perform quality assessment and benchmarking, while comparing a pair of annotations. Another shortcoming is their heavy focus on genomic data. Although through pre-processing of the data and/or slight modification of the source code, one can adjust them to process any kind of positional data, there is currently a void in, for example, solutions for working with annotations in protein sequences.

## Discussion

In this study, we present the tool SLALOM for conducting in-depth comparative and statistical analyses of annotations of continuous sequence elements in a given grouped collection of sequences, which has so far not been available for this type of analysis on this level.

Our main goals for developing the method and conducting the associated case studies were to provide a software tool for quick performance and/or correlation estimates of multiple sequence annotations, while implementing the most suitable application logic of handling ambiguities. We wanted to increase the awareness of the user for possible implications of the overlap, grouping, and averaging choices on the performance values. Finally, one of our goals was to provide the framework for in-depth analyses of differences in performance values upon changes in the chosen application logic.

SLALOM allows the user to choose the application logic of treating overlaps and duplicates within individual CSE annotations, assigning matches between annotated CSEs, treating missing values, grouping the sequences, selecting the right measures, and averaging the values to answer the question of interest.

The resulting values can vary strongly for the same input data upon changes in the chosen application logic. This phenomenon, however, does not indicate that SLALOM functions improperly or that the analysed data are of low quality. Instead, it means that the same data might answer multiple questions, delivering different answers to different questions. On the contrary, SLALOM should not be used to (over) fit the application logic in order to achieve the best performance and/or correlation values. For example, when evaluating a predictor, it is recommended to define the set of rules and principles to follow in advance, before conducting actual calculations of its performance.

The data returned by SLALOM allow further in-depth analyses of the performance of predictors, beyond the simple calculation of performance or similarity measures. These analyses are based on comparing the results obtained by running SLALOM in different modes (as illustrated by the provided case studies), or comparing the values for separate groups. For instance, one can assess the variance of performance across different groups or to look at the correlations of performance with internal group characteristics (e.g., group size or average benchmark CSE length). Such an analysis can, for example, show that a predictor works well only with relatively large group sizes (as was shown for SLiMFinder [29] in [17]) or only with long CSEs (as was shown for MEME [30]). Furthermore, due to the diverse set of proposed measures one can easily see non-optimal aspects of a predictor (e.g., high PPV in combination low TPR would imply consistent under-prediction, while high ACC with low F1 would unravel the vulnerability to the false positive paradox while working on unbalanced data). Thus, SLALOM cannot only measure the performance, but also help identify the reasons for underperformance.

The feature of flexible sequence grouping allows, among others, to compare properties of proteins between different pathways, complexes, organelles or interaction networks. The properties that can be compared include presence of specific motifs, domains or transmembrane regions.

Our method, although primarily designed to work with protein sequences, does not depend on a particular alphabet and does not need to parse the sequences themselves. Instead, it operates exclusively with sequence and group identifiers as well as integer values representing sequence lengths and border positions of the annotated elements. This level of abstraction grants two distinct advantages. First, it provides versatility. The method can be applied to any type of sequence data. In biology, this includes protein, RNA, and DNA sequences. Second, its runtime is generally not dependent on database size, total sequence length or length of annotated elements. Thus, SLALOM operates with high speed and low memory usage even on very large datasets, such as collections of genomes.

While we have shown the applicability of our tool to compare positional features in DNA or protein sequences, as well as time-series data, SLALOM itself is very versatile and can be adapted to any comparison of positional data and thus can address a multitude of questions. One could for instance apply SLALOM to evaluate the reproducibility of NGS ChIP-seq or RNA-seq data between replicates or time points/conditions. In addition, it can be used to map gene names or to associate genes with corresponding promoters. Furthermore, any type of coinciding events in time series with basic analysis of causality can be addressed, independent of the field of research.

## Conclusions

In this study, we present SLALOM, a method and its associated software package, for in-depth analysis of positional data annotating continuous sequence elements in a grouped collection of sequences.

With our examples, we show that the choice of rules and principles to treat duplications and overlaps can affect the results to a significant extent. Furthermore, we demonstrate that some statistical measures become unreliable if applied to data with some form of unbalance in it. With this study, we not only increase awareness about these statistical pitfalls, but also provide the method to detect and deal with them.

With the implemented functionality, SLALOM effectively fills the gaps in existing software solutions for comparative, statistical analysis of positional data.

## Methods

**Fig. 5:**
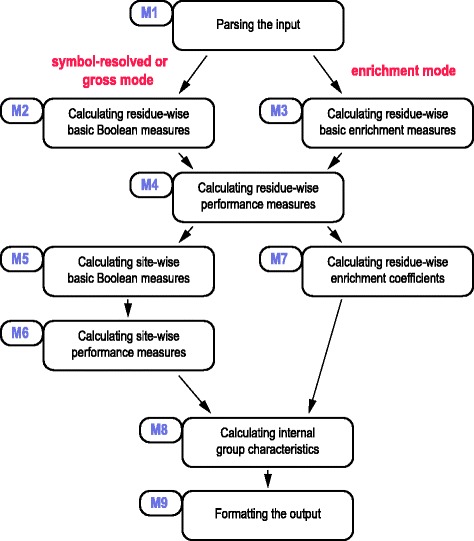
The pipeline of SLALOM. The pipeline of SLALOM is shown, which is divided into nine separate modules (M1-M9). For further information, see Main Text

SLALOM produces a table with relevant statistics describing the input data on CSE, sequence, group or dataset level and other optional output files that are derived from the input pair (e.g., as union or intersection). The method is initiated with four files: the sequence length file (sequence identifiers – SIDs – with the associated sequence lengths), two annotation files to be compared, as well as the optional group-mapping file (group identifiers – GIDs – mapped to member SIDs). The annotation files are lists of records, each of which contain: SID, GID, begin and end positions in the sequence, and optionally the CSE names. If the group-mapping file is not provided, GIDs are not read in and the whole input sequence collection is considered as a single group. If only one sequence is analysed or all the sequences have the same length, the sequence length file can be omitted too. Both annotation files must be non-empty. The desired overlap logic is set through the input options. Depending on the input options, the tool operates in one of the six modes: three count modes, each of which can be combined with two comparison modes. The modes are listed in Table 1. The input options are listed in Additional file 1: Table S1. The procedures described below represent independent modules of the pipeline. Depending on the mode, some modules may not be called. The pipeline is shown in Fig. 5.

### Module 1. Parsing the input.

The sequence length file and the group-mapping file (if provided) are checked for their validity and read into internal objects.

The following criteria must be satisfied for the parsing to complete:

- SIDs as well as GIDs cannot contain double quote signs (‘″’) as well as certain special characters
- The same SID, if it occurs more than once, cannot be assigned different length values
- A sequence length must be a positive integer
- There is at least one retained SID

The following records are ignored during the parsing:

- SIDs that do not belong to any retained group (if the group-mapping file is provided)
- GIDs that do not occur in the sequence length file
- GIDs that mapped to too few or too many retained SIDs, according to the user-defined constraints

By default, the sequence length file is read before the group-mapping file. However, if the former contains many more SIDs than the latter (for instance in case the database lists all Uniprot records and the mapping consists of only few protein complexes in a specific organism), it may be practical for performance reasons to pre-parse the group-mapping file. This option (invoked by the option ‘-preparse/--preparse_mapfile’) does not influence the output, but speeds up the calculations and reduces RAM usage considerably in case of large sequence length files.

Each of the annotation files is read into two distinct internal objects: a list of sites (sequence identifier, begin, end) and a map of residues (each residue from the database is mapped to an integer, which represents the number of sites from the given annotation traversing this residue).

The annotations are also checked for validity. The following criteria must be satisfied:

- A start position is positive and an end position does not exceed the sequence length, unless the sequences are circular; alternatively, the overflowing sites can be trimmed to fit the sequence
- An end position is no less than the corresponding start position

If an annotation record’s SID is not present in the sequence length file or the GID is not present in the group-mapping file, the record is ignored and a warning is issued.

All input files may contain duplicate records. In case of the sequence length file and the group-mapping file, duplicate entries are ignored; however, in the annotation files, all duplicates are considered, which influences the results.

### Module 2. Calculating residue-wise basic Boolean measures.

This module is called when the tool operates in the symbol-resolved or gross mode. In the symbol-resolved mode, all the residues in a particular sequence are assigned to one of four categories: PP (present-present; present at least once in both annotations), AA (absent-absent; completely absent in both annotations), AP (present exclusively in the second annotation), PA (present exclusively in the first annotation). In the gross mode, there are five categories: P1P2, P2P1, AP, PA, and AA. In the benchmarking mode, these categories correspond as following: PP to TP (true positives), AP to FP (false positives), PA to FN (false negatives), AA to TN (true negatives). In the gross mode, each residue traversed by each CSE in the first annotation is sorted to either P1P2 (if it is traversed by at least one CSE in the second annotation) or PA. Similarly, the residues from the CSEs of the second annotation are sorted to either P2P1 or AP. The AA category is the same in both symbol-resolved and gross modes. The counts are summed up and then normalized to obtain respective shares; if adjusting for sequence length or sequence-wide averaging is selected, the shares are calculated directly at the sequence level and then averaged at the group level. The shares are averaged throughout all the groups to form the basic measures PP, P1P2, P2P1, AA, AP, PA. In the symbol-resolved mode, P1P2 and P2P1 take the value of PP and PP + AA+AP + PA = 1 irrespectively of sequence lengths and group sizes. In the gross mode, it is true that min (P1P2,P2P1) + AA+AP + PA > =1; P1P2, P2P1, AP, PA ∈ [0,∞), while AA ∈ [0,1].

### Module 3. Calculating residue-wise basic enrichment measures.

This module is called when the tool operates in the enrichment mode with a user-defined positive integer ***n*** provided as parameter for the minimal occurrence of a residue in the annotations. First, for all the residues in a particular sequence, numbers of occurrences (considering all the overlaps and duplicates) in each annotation are calculated cumulatively for all CSEs. Residues that occur no less than ***n*** times in the first annotation are counted as E1 (enriched in the first). Similarly, the E2 count is defined. Residues that qualify for both E1 and E2 are additionally counted as EE (enriched-enriched), while those qualified for none are counted as NE (not enriched). Moreover, all the residues are assigned to one of three categories. To do this, the difference ***d*** between numbers of occurrences in the second and first annotations is calculated for each residue. Those with ***d*** > =***n*** are classified as RE2 (relatively enriched in the second), while those with ***d*** < = − ***n*** are classified as RE1. The rest is classified as NRE (not relatively enriched). Similarly to Module 2, the averaged measures E1, E2, EE, NE, RE1, RE2, NRE are calculated for the groups and the whole input dataset. It is true that E1 + E2-EE + NE = 1, RE1 + RE2 + NRE = 1, E1 > =EE, E2 > =EE, E1 > =RE1, and E2 > =RE2.

### Module 4. Calculating residue-wise performance measures.

This module is called in all modes; for an enrichment mode, PP = EE, AA = NE, PA = E1-EE, AP = E2-EE. These measures show, how good the annotations correspond to each other and are calculated solely on the basis of the residue-wise basic measures. The symmetric measures do not depend on the order, in which the annotations are provided. Non-symmetric measures, on the other hand, do, and thus are calculated only when the tool operates in the benchmarking mode. For details see Table 2.

The following measures are provided:

- TPR (true positive rate, a.k.a. recall/sensitivity, for the second annotation, or how good the second annotation finds the residues from the first annotation):

$$ TPR=\frac{P1P2}{P1P2+ PA} $$

- PPV (positive predictive value, a.k.a. precision, for the second annotation, or the share of residues from the second annotation, which also can be found in the first annotation):

$$ PPV=\frac{P2P1}{P2P1+ AP} $$

- SPC (specificity, a.k.a. negative rate, for the second annotation, or how good the second annotation avoids residues absent in the first annotation):

$$ SPC=\frac{AA}{AA+ AP} $$

- NPV (negative predictive value for the second annotation, or the share of residues absent in the second annotation that are also absent in the first annotation):

$$ NPV=\frac{AA}{AA+ PA} $$

- In (informedness for the second annotation):

*In* = *TPR* + *SPC* − 1

- Mk (markedness for the second annotation):

*Mk* = *PPV* + *NPV* − 1

- PC (performance coefficient for the second annotation):

$$ PC=\frac{P2P1}{P2P1+ PA+ AP} $$

- F1 (F1 score):

$$ F1=\frac{2\ast TPR\ast PPV}{TPR+ PPV}=\frac{2\ast P1P2\ast P2P1}{2\ast P1P2\ast P2P1+P2P1\ast PA+P1P2\ast AP} $$

- ACC (accuracy):

$$ ACC=\frac{PP+ AA}{PP+ PA+ AP+ AA}= PP+ AA $$

- MCC (Matthews correlation coefficient):

$$ MCC=\frac{PP\ast AA- AP\ast PA}{\sqrt{\left( PP+ PA\right)\ast \left( PP+ AP\right)\ast \left( AA+ PA\right)\ast \left( AA+ AP\right)}} $$

These calculations are conducted for each sequence group separately. If division by zero is encountered, a *nan* value is assigned. The measures are averaged across all the groups, applying simple averaging if either sequence- or group-wide averaging is chosen and micro averaging, if dataset-wide averaging is chosen. *Nan* values are by default ignored during the averaging; however, the user can also enforce counting them as zeros (for details, see Additional file 1: Table S1).

Note that there are a few small differences from the metrics used to originally evaluate the HH-MOTiF web-server in our earlier publication. First, the calculation of residue-wise PC did not consider the overlaps in the benchmark (ELM) annotation, which was implemented in the current version of SLALOM. Second, the balanced accuracy was phased out and replaced with informedness.

### Module 5. Calculating site-wise basic Boolean measures.

This module is called when the tool operates in a non-enrichment mode. For both annotations, each CSE is classified into one of two categories because a CSE either has a match in the other annotation or it has not. The classification is performed on the basis of the user-defined overlap logic. To execute this, two criteria –the ***minimal number*** of symbols and the ***minimal part*** – are tried for each CSE. For a CSE to have a match, both criteria must be satisfied, although the user can effectively switch them off by setting sufficiently low thresholds.

The ***minimal number*** of symbols criterion can be one of the following:

- There exists at least one CSE in the other annotation that has at least ***r*** (***r*** > =1) common residues with the *current* CSE (this is the criterion used by default, with ***r*** = 1)
- There exist at least ***r*** (***r*** > =1) residues contained in the CSE from the other annotation that are contained in the current CSE (the *patched* matching)

The ***minimal part*** criterion can be one of the following:

- There exists at least one CSE in the other annotation that covers at least ***p***% residues of the *current* CSE
- There exists at least one CSE in the other annotation that has at least ***p***% of residues of the *shortest* of two CSEs (the current and the matching candidate) in common
- There exists at least one CSE in the other annotation that has at least ***p***% of residues of the *longest* of two CSEs (the current and the matching candidate) in common
- There exist enough residues contained in the CSEs of the other annotation that can cover at least ***p***% of the current CSE (the *patched* matching)

For the relevant input options, see Additional file 1: Table S1. For a schematic example, see Fig. 4.

Four counts are calculated: M1 (matched for the first, number of CSEs in the first annotation that have matches in the second annotation), NM1 (not matched for the first), M2, NM2. In the benchmarking mode, NM1 becomes FN (false negatives) and NM2 becomes FP (false positives), while both M1 and M2 – depending on the metric being calculated – can be considered as TP (true positives). If no CSEs are annotated for the corresponding sequence in the annotation ***x***, then M***x*** = NM***x*** = 0. These counts are never averaged at the sequence or group level, but always simple-averaged to produce dataset-wide averages. Thus, they are not influenced by the averaging approach.

### Module 6. Calculating site-wise performance measures.

This module is called only when the tool operates in a non-enrichment mode. The calculated measures follow the similar logic, as the residue-wise ones. Note, however, that there is no analogue for AA (true negatives). Therefore, some measures cannot be calculated (for details see Table 2). To compensate for considerably lower number of measures, a new measure (positive correlation value) is introduced.

This results in the following list of site-wise measures (the counts M1, M2, NM1, NM2 are introduced in Module 5):

- Recall for the second annotation:

$$ SiteTPR=\frac{M1}{M1+ NM1} $$

- Precision for the second annotation:

$$ SitePPV=\frac{M2}{M2+ NM2} $$

- Performance coefficient for the second annotation:

$$ SitePC=\frac{M2}{M2+ NM1+ NM2} $$

- F1 score:

$$ SiteF1=\frac{2\ast M1\ast M2}{2\ast M1\ast M2+ NM1\ast M2+ NM2\ast M1} $$

- Positive correlation value (an author-defined measure):

$$ SitePCV=\frac{M1+M2}{M1+M2+ NM1+ NM2} $$

Zero division resolving and dataset-wide averaging are conducted as for the residue-wise measures (see Module 4).

### Module 7. Calculating residue-wise enrichment coefficients

This module is called only in the enrichment mode. The only measure calculated is based on the residue-wise basic enrichment measures:

- Enrichment asymmetry coefficient, or share of absolutely enriched residues that are also enriched in one of the annotations over the other (the measures E1, E2, EE, RE1, RE2 are introduced in Module 3):

$$ EAC=\frac{RE1+ RE2}{E1+E2- EE} $$

Zero division resolving and database-wide averaging are conducted as for the residue-wise performance measures (see Module 4).

### Module 8. Calculating internal group characteristics

This module is called in all modes. However, some characteristics are calculated mode-specifically.

It has the following list of characteristics:

- Number of sequences. If there are sequences in the sequence length file and the mapping file, for which no CSEs were annotated, they are still counted. If the same sequence belongs to multiple groups, it is counted the corresponding number of times.
- Number of CSEs in each annotation. Duplicated CSEs are counted separately. This measure is not calculated in the enrichment mode.
- Total length of CSEs in each annotation. Cases of overlap and duplication are treated according to the operation mode (symbol-resolved vs. gross); in the gross mode, the total length may exceed the sequence length. This measure is not calculated in the enrichment mode.
- Share of symbols belonging to the CSEs in each annotation. This share is always symbol-resolved, both in symbol-resolved and gross modes; in the enrichment mode, it is replaced with the share of enriched residues.

These characteristics are not subject to either sequence-wide or group-wide averaging. They, however, are macro-averaged dataset-wide along with the performance measures, regardless of the averaging approach selected.

### Module 9. Formatting the output

All the calculated measures according to the user settings are displayed in form of a table for each sequence, as well as for each sequence group separately. An optional detailed output file describing all the detected matches on CSE and sequence level is generated on user demand. In addition, several new annotation files are generated on demand, namely:

1. Boolean union: residues present in at least one of the input annotations
2. Boolean intersection: residues present in both input annotations
3. Boolean complement of the first: residues present exclusively in the second annotation
4. Boolean complement of the second
5. Enrichment union: residues enriched in at least one of the input annotations
6. Enrichment intersection: residues enriched in both input annotations
7. Enrichment complement of the first: residues enriched exclusively in the second annotation
8. Enrichment complement of the second
9. Relative enrichment for the first: residues that are enriched in the first annotation over the second annotation
10. Relative enrichment for the second

For the relevant input options, see Additional file 1: Table S1.

All the annotations are formatted as CSE records, i.e., neighbouring annotated residues are merged to form the lines with tab-separated GID, SID, and begin and end residues in this order.

## Additional file

Additional file 1: Contains the following supplementary information: • Command line options for SLALOM (in tabular format), • Supplementary Information for use cases 1 and 2, including the SLALOM command line options used for producing the data, • A detailed description of use case 3, • Supplementary information for the comparison of SLALOM to BioConductor and bedtools, • Supplementary references. (PDF 559 kb)
Additional file 2: Contains the python code of SLALOM, the test data required to reproduce the data presented in this manuscript, as well as the User Manual for SLALOM. (ZIP 3622 kb)
Additional file 3: Contains the R-script for addressing the three questions raised on comparison of ELM motifs in the chapter **Comparison to other CSE analysis methods.** (R 2 kb)

## Abbreviations

*CSE (continuous sequence element)*: A non-empty group of neighboring symbols in one of the input sequences.
*GID (group identifier)*: A non-empty string unequivocally relating to a specific group of input sequences.
*SID (sequence identifier)*: A non-empty string unequivocally relating to a specific input sequence.
