# Supplementary material for: SLALOM, a flexible method for the identification and statistical analysis of overlapping continuous sequence elements in sequence- and time-series data
Source: BMC Bioinformatics. 2018 Jan 26;19:24. doi: 10.1186/s12859-018-2020-x (PMC5787307; doi:10.1186/s12859-018-2020-x)
Supplement: Additional file 1: — Contains the following supplementary information: • Command line options for SLALOM (in tabular format), • Supplementary Information for use cases 1 and 2, including the SLALOM command line options used for producing the data, • A detailed description of use case 3, • Supplementary information for the comparison of SLALOM to BioConductor and bedtools, • Supplementary references. (PDF 559 kb) [file 12859_2018_2020_MOESM1_ESM.pdf]

Supplementary Material to: SLALOM, a flexible method for the identification and statistical analysis of overlapping continuous sequence elements in sequence- and time-series data

Authors: Roman Prytuliak, Friedhelm Pfeiffer, Bianca H. Habermann

Available command line options for SLALOM

Supplementary Table S1. Input command line options. Used abbreviations: SID – sequence identifier, GID – group identifier, CSE – continuous sequence element, TSV – tab-separated values.

Type	Default	Description
**Main input/output files**		
`-s, --seqlenfile`		
string	(empty)	Sequence length file. Maps SIDs to corresponding sequence lengths (or, in case of time series, start and finish points). Must be in tabular format. The file shall not be provided, if the length of all sequences is specified elsewhere
`-m, --mapfile`		
string	(empty)	Mapping file. Maps GIDs and SIDs. Must be in tabular format
`-a1, --anno1file`		
string	(required)	First/benchmark annotation file. Lists all CSEs from the first annotation, one CSE per line. Must be in tabular format, unless the simplified *GenBank* mode is activated
`-a2, --anno2file`		
string	(required)	Second (prediction) annotation file. See the option '`-a1`' for details
`-o, --outfile`		
string	(required)	Output TSV file with calculated performance measures, for each group and dataset-wide
**Simplified modes**		
`--genbank`		
bool	`false`	Activate the simplified *GenBank* mode. The annotations are expected in GenBank format. Sequence length will be read automatically
`--bed`		
bool	`false`	Activate the simplified *BED* mode. The annotations are expected in BED format
**Operation mode setup**		
`-b, --benchmarking`		
bool	`false`	Activate the benchmarking mode, i.e., treat the first annotation as benchmark and the second as prediction (default: treat the annotations symmetrically)
`-E, --enrichment_count`		
integer or string	0	Switch between symbol-resolved, gross, and enrichment

		modes. Must be a non-negative integer or 'gross'. If 0, activates the symbol-resolved mode. If >=1, activates the enrichment mode with minimal number of occurrences in CSEs of a symbol position to count it as enriched. If 'gross', activates the gross mode. See also Figure 2
**Core algorithm controls**		
-Os, --overlap_symbols		
integer	1	Minimal number of symbols required for a CSE of the query overlapping with a CSE/CSEs of the compared annotation to be counted as a match. Must be a positive integer
-Op, --overlap_part		
float	0.0	Minimal share of a query CSE required to overlap with CSE/CSEs of the other annotation to be counted as a match. Must be in range [0,1]
-Oa, --overlap_apply		
enum	shortest	The principle to apply the minimal number of symbols and the minimal part:<br>- 'shortest': to the shortest of two CSEs (one CSE from the first annotation and the other one from the second)<br>- 'longest': to the longest of two CSEs<br>- 'current': to the CSE from the annotation currently considered; CSEs from the other annotation are considered one at a time<br>- 'patched': to the CSE from the annotation currently considered; allowing multiple CSEs from the subject annotation to contribute simultaneously<br>See also Figure 4
-On, --overlap_nature		
enum	neutral	The required overlap nature of the predicted CSEs:<br>- 'neutral': count CSE overlaps regardless of the order of their start positions<br>- 'leading': only count a predicted CSE as match, if its start position is earlier or the same as that of the respective benchmark CSE<br>- 'lagging': only count predicted CSE as match, if the begin position is later or the same as that of the respective benchmark CSE<br>The default value can be changed only in the benchmarking mode and only for non-circular sequences
-a, --averaging		
enum	group	Order of averaging when calculating performance measures:<br>- 'sequence': calculate the measures for each sequence individually, then simple-average group-wide, then simple-average dataset-wide (macro-macro averaging)

		- 'group': sum the counts group-wide, then calculate the measures, then simple-average dataset-wide (micro-macro averaging)<br>- 'dataset ': sum the counts dataset-wide and calculate the measures (micro-micro averaging); for results on individual groups, apply macro averaging
-A, --adjust_for_seqlen		
bool	false	Adjust the counts for each sequence for its length, then average the adjusted values (default: sum the counts). Affects only symbol-wise measures. Not compatible with sequence-wide averaging. See also Figure 3.
**Input file format**		
-sd, --seqlenfile_delim		
char	tab	Column delimiter in the sequence length file. Allowed delimiters: space, tab, comma, dot, colon, semicolon, slash. If an empty string is passed, space will be used, multiple spaces will be collapsed and leading, as well as trailing spaces will be removed
-md, --mapfile_delim<br>-a1d, --anno1file_delim<br>-a2d, --anno2file_delim		
(see the option '-sd')		
-sh, --seqlenfile_headers		
integer	0	Number of header rows to skip in the sequence length file. Must be a non-negative integer
-mh, --mapfile_headers<br>-a1h, --anno1file_headers<br>-a2h, --anno2file_headers		
(see the option '-sh')		
-sc, --seqlenfile_colnumbers		
list of integers	(adjusted)	Comma-delimited list of column numbers (left-most column has number 1) in the sequence length file with SIDs and sequence length in that order. In case of time series, start and finish points must be provided instead of the length. The default value is adjusted according to the expected columns
-mc, --mapfile_colnumbers		
list of integers	1,2	Comma-delimited list of column numbers (left-most column has number 1) in the group-mapping file with SIDs and GIDs in that order
-a1c, --anno1file_colnumbers		
list of integers	(adjusted)	Comma-delimited list of column numbers (left-most column has number 1) in the first annotation file with CSE start positions, CSE end positions, SIDs, GIDs, and CSE names in that order (with those not provided skipped). The default value is adjusted according to the expected

		columns
-a2c, --anno2file_colnumbers		
(see the option '-a1c')		
-sq, --seqlenfile_quotes		
bool	false	Treat single quotes (apostrophes) in the sequence length file literally. By default, delimiters inside quotes (both single and double) are treated as belonging to fields and quotes themselves are ignored. Double quotes can never be part of read input; their presence in the file triggers an error, if this option is true
-mq, --mapfile_quotes<br>-a1q, --anno1file_quotes<br>-a2q, --anno2file_quotes		
(see the option '-sq')		
**Alternative input options**		
-l, --seqlen_value		
integer	0	Length of all sequences. Must be a non-negative integer. If 0, this option is ignored. If positive, the sequence length file shall not be provided; the length of every sequence is set to the specified value; all SIDs encountered in the annotations and the group-mapping file will be used. With time series options '-ts' and '-tf' shall be used instead
-ss, --single_sequence		
bool	false	Process single sequence. SIDs shall not be provided if this option is activated
-ts, --timeseries_start		
string	(empty)	Start of all time series. If empty, this option is ignored. Must be used together with the option '-tf'. For details, see the option '-l'
-tf, --timeseries_finish		
string	(empty)	Finish of all time series. For details, see the option '-ts'
-a1as, --anno1file_all_sequences		
bool	false	Consider all the CSEs from the first annotation as belonging to all the sequences. SIDs shall not be provided if this option is activated. This option cannot be selected for both annotations simultaneously
-a2as, --anno2file_all_sequences		
(see the option '-a1as')		
-a1ag, --anno2file_all_groups		
bool	false	Consider all the CSEs from the first annotation as belonging to sequences with provided SIDs in all the groups. If this option is activated, GIDs shall not be provided, although the group mapping must be provided. This option can be selected for both annotations, but it is not compatible with considering all the CSEs as belonging to all the sequences
-a2ag, --anno2file_all_groups		

(see the option '-a1ag')		
-sg, --sequences_as_groups		
bool	false	Generate respective 1-sequence groups for all the SIDs. If this option is activated, neither the group-mapping file nor GIDs shall be provided
-nOg, --non_overlapping_groups		
bool	false	Define the groups provided in the group-mapping file as non-overlapping. If this option is activated, GIDs in the annotation files shall not be provided
**Input controls**		
-n, --site_names		
bool	false	Also read in CSE names from the annotation files. This option does not affect calculations, but the names will be shown in the detailed and site-wise output files. The names can be duplicated, but cannot be empty or contain double quotes
-t, --time_unit		
enum	none	Time unit if the sequences are time series ('none' otherwise). Supported values: 'sec', 'min', 'hour', 'day'
-a1r, --anno1file_resolve		
enum	all	The rule to resolve CSE overlaps within the first annotation:<br>- 'all': leave all CSEs untouched<br>- 'first': retain only the first CSE from an overlapping group<br>- 'last': retain only the last CSE from an overlapping group<br>–'merge': merge an overlapping group to single CSE<br>If 'first' or 'last' is selected, only one CSE will be retained from the whole group, even if the first and last CSEs of the group do not overlap. For circular sequences, only 'all' and 'merge' are supported
-a2r, --anno2file_resolve		
(see the option '-a1r')		
-a1bs, --anno1file_begin_shift		
integer	0	Constant shift in symbols of CSE start positions in the first annotation; this number will be added to all the start positions. If strand detection is activated, this option affects actual end positions in the reverse strand. If frame detection is activated, the frames are not affected by this value. In the simplified *BED* mode, the start positions are additionally incremented by 1
-a2bs, --anno2file_begin_shift		
(see the option '-a1bs')		
-a1es, --anno1file_end_shift		
integer	0	Constant shift in symbols of CSE end positions in the first annotation. For details, see the option '-a1bs'

-a2es, --anno2file_end_shift		
(see the option '-a1es')		
-e, --end_overflow_policy		
enum	forbid	The rule for overflowing CSE end positions, which is required if a CSE start is non-positive or a CSE end exceeds the sequence length:<br>- 'forbid': terminate program with the error message<br>- 'trim': trim the CSE to fit the sequence; ignore, if both, begin and end exceed the sequence length and the CSE is thus completely out of the sequence scope<br>- 'ignore': ignore the CSE completely<br>- 'circular': make overflowing CSEs reappear at the other end of the sequence; the difference between the end and the begin must still be non-negative and less than the sequence length<br>For time series, 'circular' is not supported
-z, --zero_for_na		
bool	false	Treat nan values as zeros to calculate averages (default: exclude from averaging)
-min, --min_group_size		
integer	1	Minimal number of sequences in a group. Smaller groups will be ignored. Must be a positive integer
-max, --max_group_size		
integer	0	Maximal number of sequences of a group. Larger groups will be ignored. Must be a non-negative integer. If 0, the size is unlimited
-d, --detect		
enum	none	If 'strand', detect DNA strand based on the information in the file. If 'frame', in addition detect reading frames on the basis of the remainder of division by 3 of the gene start position in the corresponding strand. The frames are not adjusted by user-specified shifts (see the option '-a1bs' for details). The default value can be changed only in the simplified *GenBank* or *BED* modes
**Additional output files**		
-od, --outfile_detailed		
string	(empty)	Output file with details at the sequence and group levels
-os, --outfile_sites		
string	(empty)	Output file with site-wise statistics including match information
-ou, --outfile_union		
string	(empty)	Output TSV file with the union of the two input annotations
-oi, --outfile_intersection		
string	(empty)	Output TSV file with the intersection of the two input

		annotations
`-oc1, --outfile_complement_1`		
string	(empty)	Output TSV file with the complement of the first/benchmark annotation
`-oc2, --outfile_complement_2`		
string	(empty)	Output TSV file with the complement of the second/prediction annotation
`-ore1, --outfile_rel_enrichment_1`		
string	(empty)	Output TSV file with the sites of relative enrichment in the first/benchmark annotation
`-ore2, --outfile_rel_enrichment_2`		
string	(empty)	Output TSV file with the sites of relative enrichment in the second/prediction annotation
**Output options**		
`-osd, --outfile_sites_diff`		
enum	`all`	Limit the site-wise statistics to:<br>- ‘`all`‘: do not limit<br>- ‘`matched`‘: show only CSEs that do have a match in the other annotation under the provided criteria<br>- ‘`unmatched`‘: show only CSEs that do not have a match in the other annotation under the provided criteria<br>- ‘`discrepant`‘: show only CSEs that do not have a perfect reciprocal match in the other annotation. Not applicable, if overlap criteria are applied to ‘`patched`’<br>This option applies only in non-enrichment mode
`-c, --clean`		
bool	`false`	Produce cleaned output TSV without the commented lines. This file will contain a single header line with column names and will be ready for import into various data analysis software packages
`-sort, --sort_output`		
bool	`false`	Sort the main output file by the group names (default: sort by the order of appearance in the input). Applicable only, if groups are defined
`-sum, --calculate_sums`		
bool	`false`	Calculate sums of integer counts in addition to averages. Adds additional row in the main output table. Applicable only, if groups are defined
**Other options**		
`-preparse, --preparse_mapfile`		
bool	`false`	Pre-parse the group-mapping file before parsing the sequence length file. This option improves performance, if the number of SIDs in the sequence length file is much larger than in the mapping file

-w, --warning_level		
integer	1	Warning level:<br>- 0: switch off warnings<br>- 1: show all warnings
-q, --quiet		
bool	false	Do not print the progress in the command line

Case study 3. Influence of events on exchange rates

Sequences: time series of all minutes in a calendar year.
Groups: categories of economic events (e.g., countries of origin); each group contains a single sequence.
Benchmark annotation: directional trends in EURUSD (the underlying open-high-low-close (OHLC) data were downloaded from HistData.com).
Predictor annotation: time intervals of fixed duration from the beginning of each event (the event database was downloaded from FXStreet.com).

In this example, we treated the calendar year 2015 as a sequence of minutes (i.e., the sequence of 60*24*365=525,600 symbols). First, we marked the trends in EURUSD according to the following definition: a bullish trend is an interval encompassing as many as possible, but at least 5 consecutive M5 bars, which close higher than the previous bars, and similarly for a bearish trend. Trends that encompassed missing bars or gaps were excluded. That means, a trend of ***N*** bars has a duration of 5****N*** minutes (i.e., the length of 5****N*** symbols). There were in total 1801 trends marked. The trend time intervals were used as the benchmark. With this benchmark, we were testing, if certain events could be good predictors of approaching trends. For this purpose, we marked the time intervals of fixed duration of 30 minutes (i.e., the length of 30 symbols) following each selected event as the predictor annotation. We concentrated on site-wise TPR and FPR with the requirement of at least 50% overlap of the *current* CSE (event or trend) to match. With this logic, TPR is the share of trends, for which at least half of their duration was within the half-hour following an event, while PPV is the share of events, which for at least 15 minutes within the first half-hour after the announcement coincided with a trend. We set the groups for countries and focused on seven major economies, while events originating from other countries were implicitly filtered out. In this example, one should consider the two following issues: (1) events may occur with less than half-hour interval, and thus 'overlap' in our representation, and (2) trends may start before the event announcements. To address the former, we resolved the overlaps within the individual annotations (i.e., events in particular countries; the trends in our definition never overlap), while retaining only the last event for each overlapping group. To address the latter, we marked our predictor as 'leading', which

considers a benchmark-to-prediction CSE match only, if the start position of the predicted CSE (the event) is no greater than the start position of the benchmark CSE (the trend). As can be seen from the Supplementary Table S2, the latter requirement essentially halves the resulting PPV values. To be more precise, it multiplies them with the expected factor of 4/7. Given that the average duration of a trend is 29 minutes and therefore almost equal to the duration of an event, it can be interpreted such that events and trends are essentially independent.

Country	Number of events		Events' performance as trend predictor			
			Any begin order (option set C1)		Event is 'leading' (option set C2)	
	Total	Non-overlapping	TPR	PPV	TPR	PPV
United States	1957	1044	0.0866	0.1628	0.0489	0.0910
China	262	147	0.0133	0.1633	0.0083	0.1020
Japan	842	397	0.0316	0.1587	0.0217	0.1108
Germany	537	240	0.0272	0.2125	0.0150	0.1250
United Kingdom	828	340	0.0361	0.1971	0.0200	0.1088
France	259	154	0.0144	0.1753	0.0056	0.0714
Italy	388	183	0.0155	0.1639	0.0105	0.1093

Supplementary Table S2. Performance of economic events as a predictor of trends in EURUSD throughout the calendar year 2015

In addition to durable trends, there are also 'spikes' observed on FX markets. Spikes are very rapid and intensive movements; although their magnitude can be quite large, they only last for a few minutes or even seconds. The spikes are often associated with events (like news releases), and, as traders often end up having bigger than expected losses at the spikes (owing to slippage in stop orders), it is a common recommendation (e.g., [1]) not to trade in the wake of news releases. But how grounded are such recommendations? To shed some light on this question, we decided to look at time coincidences of events and spikes. We defined spikes as directional movements of close-to-close magnitude no lower than 0.002 USD with close-to-close on each bar no lower than 0.0005 USD in the same direction. We used M1 bars and posed no restrictions on the number of bars (i.e., it could be just one bar). We detected overall 426 spikes (or roughly 2 per trading day) with an average duration of roughly 2 minutes. We set the 'duration' of an event to 2 minutes accordingly and recorded

a match on any overlap. As a result, we found almost no time correlation of spikes and events (see Supplementary Table S3).

Country	Number of events	TPR	PPV
United States	1185	0.0094	0.0025
China	152	0.0000	0.0000
Japan	415	0.0047	0.0048
Germany	241	0.0047	0.0083
United Kingdom	341	0.0469	0.0528
France	158	0.0000	0.0000
Italy	195	0.0047	0.0103

Supplementary Table S3. Performance of economic events as a predictor of spikes in EURUSD throughout the calendar year 2015 (generated with SLALOM option set C3)

The detailed information on used command line options can be found further down in this Supplementary Material.

Supplement to the case study 1: option sets, files, and explanations.

Input files:

- `elm_seq_len_db.tsv`: database with protein sequence lengths
- `elm_instances_2016.tsv`: an ELM database dump (as of 14.03.2016)
- `hhmotif_prediction.csv`: results of the HH-MOTiF prediction, as reported in [2]

The lengths database file is a two-column TSV containing the UniprotKB identifiers and corresponding sequence lengths; if an alternative isoform is considered, the identifier is followed by dash and the isoform number. A five-line snippet from the file is shown below.

```
Q9WV93      299
P05067-4    695
O60315      1214
O14686      5537
Q9LMA8      253
```

The ELM dump is a 13-column TSV. This file was used both for grouping and as the benchmark annotation. Column 3 contains ELM class names (used as group names), column 5 contains UniprotKB identifiers, while columns 7 and 8 contain begin and end positions of the SLiMs. The rest of the columns were not used in the current analysis. The file also contains six header lines that must be skipped. The newest version of this file can be downloaded from the ELM website (elm.eu.org).

The prediction results file was generated by the command line version of the HH-MOTiF. It is a 4-column CSV. Semicolon is used as separator. The columns contain UniprotKB identifiers, ELM class names, begin and end residues of the predicted SLiMs (motif roots and motif leaves treated equally) in this order. The first five lines of the file are shown below.

```
P05132;DOC_AGCK_PIF_3;192;197
P06244;DOC_AGCK_PIF_3;335;340
Q5I6E9;DOC_AGCK_PIF_3;634;639
Q9LSF1;DOC_AGCK_PIF_3;323;328
P05412;LIG_KEPE_3;263;287
```

Command line option set A1: `python slalom.py -s elm_seq_len_db.tsv -sc 1,2 -m elm_instances_2016.tsv -mc 5,3 -mh 6 -a1 elm_instances_2016.tsv -a1c 7,8,5,3 -a1h 6 -a2 hhmotif_prediction.csv -a2c 3,4,1,2 -a2d ';' -o hhmotif_performance.tsv -min 3 -b`

This command will start the SLALOM analysis in the symbol-resolved mode, with group-wide averaging, no adjustment for sequence length, and without treating `nan`s as zeros. Supplying the additional '`-E gross`' will switch to the gross mode; the option '`-A`' switches on the adjustment for sequence length; the option '`-z`' will switch to the treating `nan`s as zeros. Table 3 in the main text contains the resulting data of all eight combinations from the corresponding columns at the bottom row of the output file.

The option set A1 is also the basis for producing the results shown in Table 4. The value in the column '***N***' has to be provided with the option '`-Os`' (1 is default); the value in the column '***P***', divided by 100, has to be provided with the option '`-Op`' (0.0 is default); the value in the column '**Applying to**' has to be provided with the option '`-Oa`'. For example, providing the additional options '`-Op 0.75 -Oa patched`' (meaning ***N***=1, ***P***=75, applying to *patched*) to the option set A1 will generate the output file with the values 0.194 and 0.304 in the bottom row of the output file in columns '`SiteTPR`' and '`SitePPV`', respectively.

Explanations of the command line options:

`-s elm_seq_len_db.tsv`	Read **s**equence length file from the file `elm_seq_len_db.tsv`
`-sc 1,2`	The file `elm_seq_len_db.tsv` contains SIDs and lengths in the **c**olumns 1 and 2 respectively
`-m elm_instances_2016.tsv`	Read group **m**apping from the file `elm_instances_2016.tsv`
`-mc 5,3`	The file `elm_seq_len_db.tsv` contains SIDs and GIDs for mapping in the **c**olumns 5 and 3 respectively
`-mh 6`	Skip the first 6 (**h**eader) rows in the group mapping file
`-a1 elm_instances_2016.tsv`	Read the first **a**nnotation (the benchmark) from the file `elm_instances_2016.tsv`
`-a1c 7,8,5,3`	The file `elm_seq_len_db.tsv` as the first annotation contains begin residues, end residues, SIDs, and GIDs in the **c**olumns 7, 8, 5, and 3 respectively
`-a1h 6`	Skip the first 6 (**h**eader) rows in the first annotation file
`-a2d ';'`	The second annotation file contains semicolon as **d**elimiter
`-min 3`	The **min**imal size of a group is 3 (i.e., discard

	GIDs that are mapped to less then 3 unique SIDs)
-b	Treat the annotations as predicted and **b**enchmark set: the first one is the **b**enchmark, while the second one is the prediction

Supplement to the case study 2: Option sets, files, and explanations

Input files:

- `N_pharaonis_annotation1.gb`: genome annotation from GenBank
- `N_pharaonis_annotation2.gb`: genome annotation from RefSeq

Both input files are in the GenBank format.

Command line option set B1: `python slalom.py --genbank -a1 N_pharaonis_annotation1.gb -a2 N_pharaonis_annotation2.gb -o scores_noframes_sr.tsv -Op 0.5 -Oa current -os mapping_noframes.txt -c`

Command line option set B2: `python slalom.py --genbank -a1 N_pharaonis_annotation1.gb -a2 N_pharaonis_annotation2.gb -o scores_noframes_gross.tsv -E gross -Op 0.5 -Oa current -os mapping_noframes.txt -c`

Command line option set B3: `python slalom.py --genbank -a1 N_pharaonis_annotation1.gb -a2 N_pharaonis_annotation2.gb -o scores_6frames_sr.tsv -d frame -Op 0.5 -Oa current -os mapping_6frames.txt -a dataset -sum -c`

Command line option set B4: `python slalom.py --genbank -a1 N_pharaonis_annotation1.gb -a2 N_pharaonis_annotation2.gb -o scores_6frames_gross.tsv -d frame -E gross -Op 0.5 -Oa current -os mapping_6frames.txt -a dataset -sum -c`

Explanations of the command line options:

`--genbank`	Start SLALOM in the simplified GenBank mode
`-a1 N_pharaonis_annotation1.gb`	The first annotation is in file `N_pharaonis_annotation1.gb`
`-a2 N_pharaonis_annotation2.gb`	The second annotation is in file `N_pharaonis_annotation2.gb`
`-d frame`	Detect reading frames of all genes
`-E gross`	Switch to the gross mode for Enrichment
`-Op 0.5`	Register a pair of genes from the different annotations as match, if the overlapping part is at least 50%

-**Oa** current	**O**verlapping criteria should be **a**pplied to the genes from the currently considered annotation
-**os** mapping_noframes.txt	Write additional **o**utput with **s**ite-wise (gene-wise) entries in the file mapping_noframes.txt
-**a** dataset	Apply dataset-wise (micro-micro) **a**veraging
-**sum**	Output **sums** in addition to averages for integer counts
-**c**	Write the '**c**lean' output scores file (without comments)

As the group mapping is not provided and the '-c' option is used, the main output files for the case without frame separation are 2-line TSVs (with one line being header), and 9-line TSVs otherwise (including both averages and sums). The column 'Nseq' contains the number 1, if there is no division by frame, and 6 otherwise. The site-wise statistics are identical for both output files in the symbol-resolved and gross modes, but show differences upon frame separation. A snippet from the file mapping_6frames.txt is shown below.

```
-1      1       1340160 1340315 NP_2772A        0       0       0       -       -
-1      1       1340385 1341170 NP_2774A        786     100     100     1340385 1341170 NP_RS06845
-1      1       1345572 1346138 NP_2782A        567     100     100     1345572 1346138 NP_RS06865
-1      1       1349697 1350671 NP_2796A        975     100     100     1349697 1350671 NP_RS06900
-1      1       1356108 1356524 NP_2808A        417     100     100     1356108 1356524 NP_RS06930
```

Column 1 contains the reading frame (e.g., -1) as sequence ID. Column 2 displays the annotation number (1 corresponds to the first annotation file, i.e., N_pharaonis_annotation1.gb in this example). Columns 3 and 4 contain the begin and end positions of the current gene, while columns 9 and 10 contain those of the best matching partner in the other annotation, or '–' if a good enough match is not found. The match is only perfect, if the values in column pairs 3 and 9 as well as 4 and 10 are identical. Column 6 indicates the number of overlapping base pairs (symbols) between the two partner genes. Column 7 and 8 contain the percentage represented by the number in column 6 with respect to the lengths of the current and the matching partner genes, respectively. Finally, columns 5 and 11 contain the gene identifiers from the input files. The latter value is empty, if no match is found. These two columns are essentially the mapping of the identifiers between the two genomes. As the number of columns and their meaning depends on the input options, the file contains the header line with the actual column names.

Supplement to the case study 3: option sets, files, and explanations

Input files:

- `eventdates2015.csv`: economic events; in the calendar year 2015, as downloaded from FXStreet.com
- `EURUSD_trends.csv`: list of detected trends in the OHLC data from HistData.com
- `EURUSD_spikes.csv`: list of detected spikes in the OHLC data from HistData.com
- `countries.txt`: list of the relevant countries

The file `eventdates2015.csv` contains 7 columns separated by commas. Column 1 contains the event announcement time, GMT. Column 3 contains the associated country. The rest of the columns were not used in this case study. A snippet from the file is shown below.

```
01/06/2015 00:30:00,Trade Balance,Australia,2,-1016.000,-877.000,
01/06/2015 01:35:00,Markit Services PMI,Japan,2,51.700,50.600,
01/06/2015 01:45:00,Caixin China Services PMI,China,2,53.400,53.000,
01/06/2015 07:45:00,Consumer Confidence,France,1,90.000,88.000,88.000
01/06/2015 08:15:00,Markit Services PMI,Spain,1,54.300,52.700,52.900
```

Both files with price movements have the same structure. They contain 3 columns separated by commas. Columns 1 and 2 contain the corresponding start and finish of a movement. Column 3 was not used in this case study.

The file `countries.txt` is the following single-column CSV:

```
United States
China
Japan
Germany
United Kingdom
France
Italy
```

Command line option set C1: `python slalom.py -t min -ts "01/01/2015 00:00:00" -tf "01/01/2016 00:00:00" -m countries.txt -mc 1,1 -a1 EURUSD_trends.csv -a1c 1,2 -a1d ',' -a1h 1 -a1as -a2 eventdates2015.csv -a2c 1,1,3 -a2h 1 -a2d ',' -a2q -a2es 30 -a2ag -a2r last -o stats_trends_neutral.tsv -b -Op 0.5 -Oa current -c`

Command line option set C2: `python slalom.py -t min -ts "01/01/2015 00:00:00" -tf "01/01/2016 00:00:00" -m countries.txt -mc 1,1 -a1 EURUSD_trends.csv -`

`a1c 1,2 -a1d ',' -a1h 1 -a1as -a2 eventdates2015.csv -a2c 1,1,3 -a2h 1 -a2d ',' -a2q -a2es 30 -a2ag -a2r last -o stats_trends_leading.tsv -b -Op 0.5 -Oa current -On leading -c`

Command line option set C3: `python slalom.py -t min -ts "01/01/2015 00:00:00" -tf "01/01/2016 00:00:00" -m countries.txt -mc 1,1 -a1 EURUSD_spikes.csv -a1c 1,2 -a1d ',' -a1h 1 -a1as -a2 eventdates2015.csv -a2c 1,1,3 -a2h 1 -a2d ',' -a2q -a2es 2 -a2ag -a2r last -o stats_spikes.tsv -b -On leading -c`

Explanations of the command line options:

`-t min`	Minute is used as the **t**ime unit
`-ts "01/01/2015 00:00:00"`	**S**tart of all time series (for all the countries) is midnight of January 1st, 2015
`-tf "01/01/2016 00:00:00"`	**F**inish of all time series (for all the countries) is midnight of January 1st, 2016
`-m countries.txt`	The country group **m**apping is in the file `countries.txt`
`-mc 1,1`	Let each country be a group on its own by reading the country and the group names from the same column (**c**olumn 1) of the file
`-a1 EURUSD_trends.csv`	Read the first **a**nnotation (trends) from the file `EURUSD_trends.csv`
`-a1c 1,2`	Read the starts and ends of the trends from the **c**olumns 1 and 2 of the file, respectively; SIDs and GIDs are not provided because the option '`-a1as`' is used
`-a1d ','`	Column **d**elimiter in the first annotation file is comma
`-a1h 1`	Skip 1 (**h**eader) row at the beginning of the first annotation file
`-a1as`	All time intervals in the first annotation belong to **a**ll **s**equences (i.e., considered for every country); do not read SIDs and GIDs from the annotation file itself
`-a2 eventdates2015.csv`	Read the second **a**nnotation (events) from the file `eventdates2015.csv`
`-a2c 1,1,3`	Read the starts and 'ends' of the events from the same **c**olumn 1 of the file; read SIDs (country names) from **c**olumn 3; GIDs are not provided because of the option '`–a2ag`'
`-a2q`	Read in single **q**uotes from the second annotation file literally
`-a2ag`	All time intervals in the second annotation

	belong to **a**ll **g**roups, which contain the corresponding SID (i.e., to all country groups that contain this country)
`-a2es 30`	The **e**nds' **s**hift in the second annotation is 30 symbols forward (i.e., events 'end' 30 minutes after they are announced)
`-a2r last`	**R**esolve 'overlaps' within the second annotation by considering only the last event from an 'overlapping' group
`-b`	Treat the first annotation (trends) as the **b**enchmark, while the second (events) – as prediction
`-Op 0.5`	Register a trend-event pair as match, if their **o**verlapping time **p**art is at least 50%
`-Oa current`	**O**verlapping criteria should be **a**pplied to the event from the currently considered annotation: to the benchmark while calculating TPR; and to the prediction if calculating PPV
`-On leading`	Consider only those pairs as as matches, in which the event starts no later than the trend: the **o**verlap **n**ature is leading
`-c`	Write the **c**lean output scores file, without comments and the average values

As the '`-c`' option is used, the output files are 8-line TSVs (with one line being a header) with 29 columns. The column '`SiteNP`' contains the number of sites in the prediction annotation, i.e., number of events considered. To obtain the total number of events, including the overlapping ones, one should not provide the option '`-a2r last`'. Similarly, the column '`SiteNB`' contains the number of trends or spikes. Its value is equal for all countries, as all movements are always considered. The columns '`SiteTPR`' and '`SitePPV`' were used to produce the data for Tables 5 and 6. The column '`Nseq`' contains the number 1, because there is a single country in each country 'group'. Alternatively, one could group the countries by region: Germany, France, and Italy under 'EU', and China and Japan under 'Asia' (as in the provided file `country_groups.tsv`, which is to be used with the option '`-mc 2,1`'). In this case one gets only 4 lines of data instead of 7, each line containing the statistics on the whole group.

Supplement to the comparison to other CSE analysis methods

Input files:

- `elm_instance_2016.tsv`: dump of the ELM database as of 14.03.2016
- `elm_instance_2017.tsv`: dump of the ELM database as of 14.10.2017
- `uniprot-all.tsv`: list of all the Uniprot records mapping the identifiers (primary accessions) to the sequence lengths; owing to the large size, this file is not included, but its recent version can be freely obtained from Uniprot
- `uniprot-relevant.tsv`: same as `uniprot-all.tsv`, but with records mentioned in neither of the two compared ELM database versions removed

The two ELM dumps have the same structure: 13-column TSVs with 6 header rows. For the current analysis, we consider only four columns: column 3 with the ELM class identifiers, column 5 with the Uniprot identifiers, and columns 7 and 8 with the start and end motif positions, respectively.

The Uniprot files are 2-column TSVs with a header row.

Command line option set D1: `python slalom.py -s uniprot-all.tsv -sc 1,2 -sh 1 -m elm_instances_2016.tsv -mc 5,3 -mh 6 -a1 elm_instances_2016.tsv -a1c 7,8,5,3 -a1h 6 -a2 elm_instances_2017.tsv -a2c 7,8,5,3 -a2h 6 -o output_comparison_questions1_2.tsv -b -Op 1.0 -Oa longest -a dataset`

Command line option set D2: `python slalom.py -s uniprot-all.tsv -sc 1,2 -sh 1 -m elm_instances_2016.tsv -mc 5,3 -mh 6 -a1 elm_instances_2016.tsv -a1c 7,8,5,3 -a1h 6 -a2 elm_instances_2017.tsv -a2c 7,8,5,3 -a2h 6 -o output_comparison_question3.tsv -b -Op 0.5 -Oa current -a dataset`

We wanted to address the questions formulated in the Main Text using publicly and readily downloadable files. Next to database dumps from the ELM resource, sequence length information is needed, which can be retrieved from the Uniprot database. As Uniprot only lists canonical isoforms, we decided to ignore motifs in sequences of non-canonical isoforms.

Furthermore, as some Uniprot identifiers mentioned in ELM were already phased out by Uniprot, we also ignored motifs from those sequences.

The choices we made on micro or macro averaging for comparison between SLALOM and BioConductor are for demonstration purposes only and are not the only ones possible. We rather demonstrate that both BioConductor and SLALOM are flexible in this regard and can be adjusted for the averaging method required.

As the benchmarking mode is used, motif residues in the first annotation are viewed as golden standard (relevant) elements, and the ones in the second annotation are viewed as predicted (selected) elements. Consequently, the column '`Rel.`' ('relevant') of the output file '`output_comparison_questions1_2.tsv`' contains the macro-averaged share of total protein length covered by the motif instances for all the proteins marked as containing motifs from a specific ELM class. The average (the bottom row) in this column is the simple average for all classes. Similarly, the column '`Sel.`' ('selected') contains the corresponding share for the second annotation. These averages are the answer for question 1 from the Main Text. In the provided BioConductor R script, only the former value is calculated.

The options '`-Op 1.0 -Oa longest`' literally register a matching motif between the two annotations, if the overlap is 100% of the length of the longest motif. This is only possible, if the match is exact on residue level. On the other hand, the options '`-Op 0.5 -Oa current`' are more relaxed, as they require only 50% coverage for the currently considered motif instance, which may be the shorter one. The share of motif instances in the first annotation having an exact match in the second one – the answer for question 2 from Main Text – can be found in the bottom row ('`Average`') and the column '`SiteTPR`' of the output file '`output_comparison_questions1_2.tsv`'. The corresponding share of motif instances covered by at least 50% of their length by a specific instance form the second annotation – the answer to the question 3 – can be found at the same place in the output file '`output_comparison_question3.tsv`'. In this example, micro-averaging is done at all levels using the option '`-a dataset`'.

Supplementary References

1. Sether L, Wasendorf RR. Forex Trading. 2009;

2. Prytuliak R, Volkmer M, Meier M, et al. HH-MOTiF: de novo detection of short linear motifs in proteins by Hidden Markov Model comparisons. Nucleic Acids Res. 2017;45:W470-W477
