# Supplementary material for: SLALOM, a flexible method for the identification and statistical analysis of overlapping continuous sequence elements in sequence- and time-series data
Source: BMC Bioinformatics. 2018 Jan 26;19:24. doi: 10.1186/s12859-018-2020-x (PMC5787307; doi:10.1186/s12859-018-2020-x)
Supplement: Additional file 2: — Contains the python code of SLALOM, the test data required to reproduce the data presented in this manuscript, as well as the User Manual for SLALOM. (ZIP 3622 kb) [file 12859_2018_2020_MOESM2_ESM.zip › User_manual.pdf]

SLALOM (v2.1.4) User Manual

Content

1. About SLALOM 1
2. Download and Installation 1
3. Input Files 2
4. Analysis of Grouped Sequences 3
5. The Main Output File 4
6. The Operating Modes 5
7. Annotations in GenBank Format 6
8. Annotations in BED Format 7
9. Averaging Approaches 8
10. Resolving Overlaps within the Annotations 10
11. Overlap Criteria between the Annotations 12
12. Detailed, Site-wise Statistics as Output 14
13. Mapping of Site Names/Identifiers between the Annotations 15
14. Circular Sequences as Input 17
15. Custom Tabular Files as Input 18
16. Time Series as Input 21
17. Summary of Available Command Line Options for SLALOM 21

1. About SLALOM

SLALOM (StatisticaL Analysis of Locus Overlap Method) is a standalone software tool for analysis of positional data. Namely, it compares two distinct lists of site intervals (hereafter – sites; in the associated publication referred to as continuous sequence elements - CSEs) in a given set of sequences to evaluate the overlap quality according to rules set by the user. The sites are presented as sequence coordinates, with the start and end positions, while the sequences can be of any nature (e.g., alphabetical or time series). This manual is written as a tutorial, each section describing a specific aspect; for the complete list of available command line options see the last section. Please, read the associated publication [Prytuliak, et al., SLALOM, a flexible method for the identification and statistical analysis of overlapping continuous sequence elements in sequence- and time-series data, Publication pending] for detailed explanations of the method, specific cases, and comparison with the other tools.

2. Download and Installation

SLALOM is open-source software distributed under the GPLv3 license; its latest version can be freely downloaded from the GitHub repository: https://github.com/BCF-calanques/SLALOM. Alternatively, if `git` is installed, type in the terminal:

```
>git clone -b master --single_branch https://github.com/BCF-calanques/SLALOM --depth 1
```

This will generate the directory `SLALOM/` with the source code in the subdirectory `SLALOM/src/` and all the mentioned test files in the subdirectory `SLALOM/test_data/`.

SLALOM is a command-line tool written in Python. It is cross-platform and does not require additional installation beyond a Python interpreter of version 3.5 or later (https://www.python.org/downloads/) and `numpy` library for scientific computing (https://www.numpy.org). To install `numpy` from the command line using Python package manager, type:

```
>pip install numpy
```

That's all! To use SLALOM, simply run `slalom.py`. For example:

```
>python SLALOM/src/slalom.py --help
```

3. Input Files

`slalom.py` expects four input files: two annotations (i.e., lists of sites in given sequences), the sequence length table and the group-mapping table, the last two being optional:

```
>python slalom.py [options] -a1 <first_annotation_file> -a2 <first_annotation_file> [-s <sequence_length_table>] [-m <group_mapping_table>] -o <output_file>
```

The main input files are the two site annotations to compare. In the basic case, they contain 3 columns: the sequence identifier (SID) the start and the end positions of the sites. For example:

```
>cat test_anno1.tsv
seqA  6     10
seqA  8     12
seqA  9     18
seqB  15    25
>cat test_anno2.tsv
seqA  5     11
seqA  7     10
seqA  9     15
seqB  15    19
seqB  21    25
```

By default, the numeration of symbol positions in a sequence starts from 1. Both start and end positions are considered included. That means that the length of a site that starts at 15 and ends at 25 is 11 symbols. The sites can overlap, contain or be duplications of each other.

The annotation files must be provided with the options `-a1`/`--anno1file` and `-a2`/`--anno2file`.

To complete the input, the user needs to specify the sequence length (say, all the sequences are 50 symbols long) with the option `-l`/`--seqlen_value` and specify the output file with the option `-o`/`--outfile`:

```
>python slalom.py -a1 test_anno1.tsv -a2 test_anno2.tsv -l 50 -o output.tsv
```

Alternatively, the sequence lengths can be provided in the file using the option `-s`/`--seqlenfile`. This is the only option, if the sequences have different lengths. The file should contain two columns – the SIDs and their lengths:

```
>cat test_seqlenfile1.tsv
seqA  50
seqB  30
seqC  45
seqD  60
>python slalom.py -a1 test_anno1.tsv -a2 test_anno2.tsv -s test_seqlenfile1.tsv -o
output.tsv
```

4. Analysis of Grouped Sequences

Sometimes the sequences and sites are grouped into categories and a separate analysis within each category is required. To enable sequence grouping, the user should provide the file with sequence-to-group mapping. The file should contain two columns: SIDs and respective group identifiers (GIDs). The same sequence can belong to multiple groups; a group can contain arbitrary number of sequences:

```
>cat test_groupmapping.tsv
seqA  group0
seqB  group0
seqB  group1
seqC  group1
seqD  group2
```

For multi-group analysis, the user should also specify the group for each site. An extra column should be added to the annotation files:

```
>cat test_anno3.tsv
seqA  group0      8     15
seqA  group0      21    25
seqB  group0      6     9
seqB  group1      8     11
seqC  group1      18    24
seqD  group2      1     9
seqD  group2      5     12
>cat test_anno4.tsv
seqB  group1      8     12
seqC  group1      11    16
seqD  group2      5     10
```

Note that in this example, the two sites in `seqB` in `test_anno3.tsv` are no longer considered overlapping for the analysis, as they belong to different groups.

The group-mapping table must be provided with the option `-m`/`--mapfile`:

```
>python slalom.py -a1 test_anno3.tsv -a2 test_anno4.tsv -s test_seqlenfile1.tsv -m
test_groupmapping.tsv -o output.tsv
```

An input annotation with the grouping information can be reused as the group-mapping table (duplicating records are no problem):

```
>python slalom.py -a1 test_anno3.tsv -a2 test_anno4.tsv -s test_seqlenfile1.tsv -m
test_anno3.tsv -o output.tsv
```

The four input files specified by the -a1, -a2, -s, and -m options provide flexibility in general cases. However, in many practical situations, there is some additional information available allowing for simpler input. For example, when the groups do not overlap, a site can belong to one group only, and therefore it is no longer necessary to specify GIDs in the annotation files. This can be indicated by the option -nOg/--non_overlapping_groups. Furthermore, if each sequence forms its own unique group, the user can activate the option -sg/--sequences_as_groups and skip the group-mapping file. These and other options useful in simplified cases are discussed in more detail further down in this manual.

5. The Main Output File

The main output file begins with several commented (i.e., starting with #) lines, containing information about the input command line as well as about the statistics calculated:

```
# This file was generated at 2017-01-01 00:00:01 with SLALOM
# Command line options (unquoted and unescaped): -a1 test_anno3.tsv -a2 test_anno4.tsv
-s test_seqlenfile1.tsv -m test_anno3.tsv -o output.tsv
# The following statistics have been calculated:
#     Nseq: Number of sequences
#     P1: Fraction of symbols present in the first annotation
#     P2: Fraction of symbols present in the second annotation
#     PP: Fraction of symbols present in both annotations
#     AP: Fraction of symbols absent in the first annotation but present in the second
#     PA: Fraction of symbols present in the first annotation but absent in the second
#     AA: Fraction of symbols absent in both annotations
#     ACC: Symbol-wise accuracy
#     MCC: Symbol-wise Matthews correlation coefficient
#     F1: Symbol-wise F1 score
#     SiteN1: Number of sites in the first annotation
#     SiteN2: Number of sites in the second annotation
#     SiteN1m: Number of matched sites in the first annotation
#     SiteN2m: Number of matched sites in the second annotation
#     SiteL1: Total length of sites in the first annotation
#     SiteL2: Total length of sites in the second annotation
#     SiteF1: Site-wise F1 score
#     SitePCV: Site-wise positive correlation value
# All the provided dataset-wide averages are macro-averages of the group-wide metrics
```

This block is followed by the table with the first row being the header containing the column names. The commented block is helpful at the first usages of SLALOM. However, for easier further processing of the output, it can be suppressed with the option -c/--clean:

```
>python slalom.py -a1 test_anno3.tsv -a2 test_anno4.tsv -s test_seqlenfile1.tsv -m
test_groupmapping.tsv -o output.tsv -c
```

If this option is provided, only the actual table (with the header) row is returned:

```
>cat output.tsv
Group   Nseq    P1      …  SiteN1  SiteN2  SiteN1m SiteN2m SiteL1  SiteL2  …
group0  2       0.2125  …  3       0       0       0       17      0       …
group1  2       0.1467  …  2       2       1       1       11      11      …
group2  1       0.2000  …  2       1       2       1       12      6       …
Average 1.6667  0.1864  …  2.3333  1.0000  1.0000  0.6667  13.333  5.6667  …
```

For metrics like total site length, the sum may be more informative than the average. The option -sum/--calculate_sums will add the corresponding row to the output:

```
>python slalom.py -a1 test_anno3.tsv -a2 test_anno4.tsv -s test_seqlenfile1.tsv -m
test_groupmapping.tsv -o output.tsv -c -sum --quiet
>cat output.tsv
```

Group	Nseq	P1	…	SiteN1	SiteN2	SiteN1m	SiteN2m	SiteL1	SiteL2	…
group0	2	0.1967	…	3	0	0	0	17	0	…
group1	2	0.1444	…	2	2	1	1	11	11	…
group2	1	0.2000	…	2	1	2	1	12	6	…
Average	1.6667	0.1804	…	2.3333	1.0000	1.0000	0.6667	13.333	5.6667	…
Sum	5		…	7	3	3	2	40	17	…

Note that sequences belonging to multiple groups are counted the corresponding numbers of times.

6. The Operating Modes

SLALOM supports three different approaches of handling overlapping sites in one annotation: the symbol-resolved, gross, and enrichment modes. In addition, the annotations can be treated symmetrically – if they have similar origins and reliability – or the first annotation can be used as benchmark to evaluate the second one: the equal and benchmarking modes.

In the output file shown in the previous section, one can see that the total length of sites in the first annotation (the column `SiteL1`) for `group2` equals to 12 or the fraction of 20% (the column `P1`). This means that implicit merging of the two overlapping sites (`1`-`9` and `5`-`12`) happened. Note, however, that the number of sites in the first annotation (the column `SiteN1`) still equals to 2 and the site-based statistics are calculated accordingly. This behavior is termed 'symbol-resolved mode' and is applied by default.

Sometimes, however, there is a need to count the symbols as many times as they occur in a given annotation, i.e., as they are traversed by a site. This behavior is termed 'gross mode' and is invoked by providing the value `gross` with the option `-E`/`--enrichment_count`:

```
>python slalom.py -a1 test_anno3.tsv -a2 test_anno4.tsv -s test_seqlenfile1.tsv -m
test_groupmapping.tsv -o output.tsv -E gross -c --quiet
>cat output.tsv
```

Group	Nseq	…	SiteN1	SiteN2	SiteN1m	SiteN2m	SiteL1	SiteL2	…
group0	2	…	3	0	0	0	17	0	…
group1	2	…	2	2	1	1	11	11	…
group2	1	…	2	1	2	1	17	6	…
Average	1.6667	…	2.3333	1.0000	1.0000	0.6667	15.000	5.6667	…

This time, the total length of sites in the first annotation is 17; the fraction of symbols is not calculated in the gross mode, as the metric could exceed 1 now.

In some other situations, the user may want to consider only those positions that are traversed by a certain minimal number of sites – the 'enrichment mode'. This number must be provided with the option `-E`/`--enrichment_count`. In the enrichment mode, the calculated metrics have slightly different names. For example, the fraction of symbols *present* in the first annotation (the column `P1`) becomes the fraction of symbols *enriched* (the column `E1`). If it is set, for example, to 2, this results in considering only those positions that belong at least to 2 sites. In the provided example, there are no such positions in the first annotation for `group0` and `group1`; for `group2`, there are only 5 positions (5-9 in `seqD`) for 60 symbols, which results in the fraction of 0.0833:

```
>python slalom.py -a1 test_anno3.tsv -a2 test_anno4.tsv -s test_seqlenfile1.tsv -m test_groupmapping.tsv -o output.tsv -E 2 -c --quiet
>cat output.tsv
Group    Nseq    E1      …
group0   2       0.0000  …
group1   2       0.0000  …
group2   1       0.0833  …
Average 1.6667   0.0278  …
```

In addition to different strategies of resolving the overlaps within one annotation, the annotations can have different relations to each other. One scenario is comparing annotations, which relate to the same type of sites (e.g., genes in both annotations), come from similar origins, and have comparable quality. This scenario is called 'equal mode' and is assumed by default. However, when sites in different annotations represent different types of regions (e.g., genes and promoters) or have significantly different reliability (e.g., experimentally verified and predicted), a different scenario becomes relevant. This scenario is called 'benchmarking mode' and is assumed if the option `-b`/`--benchmarking` is provided:

```
>python slalom.py -a1 test_anno3.tsv -a2 test_anno4.tsv -s test_seqlenfile1.tsv -m test_groupmapping.tsv -o output.tsv -b -c
```

The change from the equal to benchmarking mode involves both, naming conventions, as well as enabling calculation of additional performance measures. The names are chosen to highlight the non-symmetry in the benchmarking mode: the 'first annotation' becomes 'benchmark', the 'second annotation' becomes 'prediction', the metric 'present in both annotations' becomes 'true positive', etc. Nevertheless, changes in names do not change the calculations: the values of the returned metrics are equivalent in both modes. In the equal mode, however, only symmetric performance measures – those unaffected by flipping the annotation files – are calculated. Accuracy (ACC) and F1 score are examples of symmetric measures. In the benchmarking mode, additional, non-symmetric measures are calculated, for example, specificity (SPC) and precision (PPV). Thus, the equal mode is effectively just a subset of the benchmarking mode and brings only small advantages with respect to speed and output file size; however, it was implemented to prevent the user from misinterpreting certain metrics in the case of symmetric annotations.

The operating modes should not be confused with the simplified modes for easier processing of certain file formats (see next two sections) and can be used independently of those.

7. Annotations in GenBank Format

Although SLALOM works primarily with tabular input, it has a special simplified mode to compare genome annotations in GenBank format. This mode works under the assumption that both annotations are of the same sequence. The sequence length is automatically read from the files and must not be provided separately. To start the program in the GenBank mode, the user needs to provide the `--genbank` option:

```
>python slalom.py --genbank -a1 N_pharaonis_annotation1.gb -a2 N_pharaonis_annotation2.gb -o comparison_stats.tsv -c
```

In the given example, there will be one data row in the table, as only one sequence (although perhaps a very long one) is analyzed:

```
>cat comparison_stats.tsv
Nseq     …  SiteN1   SiteN2   SiteN1m SiteN2m SiteL1   SiteL2   SiteF1   SitePCV
```

```
1         …  2694     2608     2622     2602     2359033 2336204 0.9853   0.9853
```

However, the user can analyze each strand or reading frame separately. For this, the value `strand` or `frame` respectively must be provided with the option `-d`/`--detect`:

```
>python slalom.py --genbank -a1 N_pharaonis_annotation1.gb -a2
N_pharaonis_annotation2.gb -o comparison_stats.tsv -c -d frame -sum
```

Now, the output file contains eight rows: the header, statistics for each of the six reading frames, and the bottom lines with the averages and sums:

```
>cat comparison_stats.tsv
Frame    Nseq     …  SiteN1   SiteN2   SiteN1m  SiteN2m  SiteL1   SiteL2   SiteF1   SitePCV
+1       1        …  475      458      453      453      419436   410952   0.9711   0.9711
+2       1        …  473      460      459      459      416979   411405   0.9839   0.9839
+3       1        …  459      441      439      440      400059   394497   0.9766   0.9767
-1       1        …  456      438      436      436      384372   382003   0.9754   0.9754
-2       1        …  382      376      372      372      344138   339013   0.9815   0.9815
-3       1        …  449      435      430      431      402931   401475   0.9740   0.9740
Average  1.0000   …  449.00   434.67   431.50   431.83   394652   389891   0.9771   0.9771
Sum      6        …  2694     2608     2589     2591     2367915  2339345
```

From this table, one can see that there are a total of 475 genes in the reading frame `+1` of the first annotation but only 458 genes for the second annotation. Moreover, 453 genes in each annotation overlap with a gene from another annotation (at this stage, it is not possible to conclude that all the matches are reciprocal). The total length of the genes is 419,436 bp for the first annotation and 410,952 bp for the second annotation. If for some reason, there are any overlapping genes annotated, they are merged for this residue calculation, as SLALOM is operating in the default symbol-resolved mode. Also because of the merging, the total gene length without the frame separation (2,359,033 bp) is less than the sum of the frame-wise gene lengths for the first annotation (2,367,915 bp).

8. Annotations in BED Format

SLALOM offers a simplified mode to process annotations in BED format. To use it, the user has to provide the option `--bed`, as well as the two annotation files, the sequence length table, and the output file:

```
>cat test_bed1.bed
chr1    0       10
chr1    15      25
chr1    40      50
chr2    12      27
>cat test_bed2.bed
chr1    0       12
chr1    25      42
>cat test_seqlenfile2.tsv
chr1    50
chr2    40
>python slalom.py --bed -a1 test_bed1.bed -a2 test_bed2.bed -s test_seqlenfile2.tsv -o
output.tsv -c
```

Note that although SLALOM by default counts residues starting from 1 with both start and end residues being included, it is automatically adjusted to the BED format specifications, which require counting starting from 0 with only the start being included.

The default output table contains only the summary line:

```
>cat output.tsv
Nseq     P1       …  SiteN1   SiteN2   SiteN1m  SiteN2m  SiteL1   SiteL2   SiteF1   SitePCV
2        0.5000   …  4        2        2        2        45       29       0.6667   0.6667
```

As the BED format does not explicitly specify group identifiers, grouping of sequences is not possible in this simplified mode. However, with the option `-sg`/`--sequences_as_groups`, each sequence will be treated as a group on its own and the statistics for each sequence will be printed separately:

```
>python slalom.py --bed -a1 test_bed1.bed -a2 test_bed2.bed -s test_seqlenfile2.tsv -o output.tsv -c -sg -sum --quiet
>cat output.tsv
Seq.    Nseq    P1       …  SiteN1   SiteN2   SiteN1m  SiteN2m  SiteL1   SiteL2   SiteF1   …
chr1    1       0.6000   …  3        2        2        2        30       29       0.8000   …
chr2    1       0.3750   …  1        0        0        0        15       0        nan      …
Average 1.0000  0.4875   …  2.0000   1.0000   1.0000   1.0000   22.500   14.500   0.8000   …
Sum     2                …  4        2        2        2        45       29                …
```

If the BED files contain strand information, the statistics for each strand separately can be calculated:

```
>cat test_bed3.bed
chr1    0       10      featA   0       +
chr1    15      25      featB   0       -
chr1    40      50      featC   0       +
chr2    12      27      featD   0       -
>cat test_bed4.bed
chr1    0       12      featE   0       +
chr1    25      42      featF   0       -
>python slalom.py --bed -a1 test_bed3.bed -a2 test_bed4.bed -s test_seqlenfile2.tsv -o output.tsv -c -d strand -sum --quiet
>cat output.tsv
Seq.    Nseq    P1       …  SiteN1   SiteN2   SiteN1m  SiteN2m  SiteL1   SiteL2   SiteF1   …
chr1+   1       0.4000   …  2        1        1        1        20       12       0.6667   …
chr1-   1       0.2000   …  1        1        0        0        10       17       nan      …
chr2-   1       0.3750   …  1        0        0        0        15       0        nan      …
Average 1.0000  0.3250   …  1.3333   0.6667   0.3333   0.3333   15.000   9.6667   0.6667   …
Sum     3                …  4        2        1        1        45       29                …
```

Note that in case of strand or frame detection, the option of treating sequences as groups is activated automatically.

9. Averaging Approaches

In the previous two sections, one could have noticed that some metrics – for example, the site-wise F1 score – change their value merely on turning on and off a sequence grouping option. This is not a bug in SLALOM, but a consequence of differences between micro and macro averaging. With micro averaging, all the symbol and site counts are summed up before the performance metrics formulas are applied. With macro averaging, the formulas applied first and the results are simple-averaged to produce the displayed metrics. Averaging is applied twice – to the sequence data at the group level as well as to the group data at the dataset level – and is regulated through the option `-a`/`--averaging`. Providing value `sequence` will trigger macro averaging on both levels, while providing `dataset` will trigger micro averaging; the default value `group` sets micro averaging at the group-level and macro averaging at the dataset-level.

Thus, unless dataset-wise averaging is applied, the average value for a column is the simple average of the corresponding group-wide values (`nan`s are ignored):

```
>python slalom.py --bed -a1 test_bed3.bed -a2 test_bed4.bed -s test_seqlenfile2.tsv -o output.tsv -c -d strand -sum --benchmarking --quiet
>cat output.tsv
Seq.    Nseq    …  SiteNB  SiteNP  SiteNBm SiteNPm SiteLB  SiteLP  SiteTPR SitePPV …
chr1+   1       …  2       1       1       1       20      12      0.5000  1.0000  …
chr1-   1       …  1       1       0       0       10      17      0.0000  0.0000  …
chr2-   1       …  1       0       0       0       15      0       0.0000  nan     …
Average 1.0000  …  1.3333  0.6667  0.3333  0.3333  15.000  9.6667  0.1667  0.5000  …
Sum     3       …  4       2       1       1       45      29                      …
```

In the benchmarking mode, the site-wise TPR (true positive rate; a.k.a. recall or sensitivity) is the ratio of the number of matched benchmark sites (`SiteNBm`) to the total number of benchmark sites (`SiteNB`); the site-wise PPV (positive predictive value; a.k.a. precision) is the ratio of the number of matched predicted sites (`SiteNPm`) to the total number of predicted sites (`SiteNP`). These numbers are calculated for each group and then averaged.

Under dataset-wise averaging, however, the respective calculations are performed on the summed-up numbers of sites:

```
>python slalom.py --bed -a1 test_bed3.bed -a2 test_bed4.bed -s test_seqlenfile2.tsv -o output.tsv -c -d strand -sum -b -a dataset --quiet
>cat output.tsv
Seq.    Nseq    …  SiteNB  SiteNP  SiteNBm SiteNPm SiteLB  SiteLP  SiteTPR SitePPV …
chr1+   1       …  2       1       1       1       20      12      0.5000  1.0000  …
chr1-   1       …  1       1       0       0       10      17      0.0000  0.0000  …
chr2-   1       …  1       0       0       0       15      0       0.0000  nan     …
Average 1.0000  …  1.3333  0.6667  0.3333  0.3333  15.000  9.6667  0.2500  0.5000  …
Sum     3       …  4       2       1       1       45      29                      …
```

Only one site out of four was recalled for the whole dataset, and therefore the site-wise TPR is now 0.25. The site-wise PPV remains at 0.5, although it would also change for a different dataset.

Another aspect of averaging is sequence length adjustment. Depending on the underlying question, a site of length 5 in a sequence of length 100 may be weighted differently than another site of length 5 in a sequence of length 25. As a result, it may happen that, if only one of such two sites in a benchmark annotation was recalled/predicted correctly in another annotation, the user would like to see different values depending on which site exactly is predicted correctly. Under the default settings, the performance metrics are not adjusted for the sequence length, and therefore the results are equivalent for both situations:

```
>cat test_seqlenfile3.tsv
seqA    100
seqB    25
>cat test_anno_benchmark.tsv
seqA    21      25
seqB    6       10
>cat test_anno_prediction1.tsv
seqA    21      25
seqB    16      20
>cat test_anno_prediction2.tsv
seqA    41      45
seqB    6       10
>python slalom.py -a1 test_anno_benchmark.tsv -a2 test_anno_prediction1.tsv -s test_seqlenfile3.tsv -o output.tsv -c -b --quiet
```

```
>cat output.tsv
Nseq    …  TP      FP      FN      …  TPR     PPV     SPC     …  SiteTPR SitePPV …
2       …  0.0400  0.0400  0.0400  …  0.5000  0.5000  0.9565  …  0.5000  0.5000  …
>python slalom.py -a1 test_anno_benchmark.tsv -a2 test_anno_prediction2.tsv -s
test_seqlenfile3.tsv -o output.tsv -c -b --quiet
>cat output.tsv
Nseq    …  TP      FP      FN      …  TPR     PPV     SPC     …  SiteTPR SitePPV …
2       …  0.0400  0.0400  0.0400  …  0.5000  0.5000  0.9565  …  0.5000  0.5000  …
```

The output changes accordingly, if the option `-A`/`--adjust_for_seqlen` is provided. It acts like a variation of sequence-wise (macro) averaging at the group level. While by default the sequence lengths and TP (true positive), FP (false positive), etc. counts are summed-up for the whole group and then used to calculate the respective fractions (columns `TP`, `FP`, etc.), the fractions are calculated for each sequence and then simple-averaged when adjusting for sequence length:

```
>python slalom.py -a1 test_anno_benchmark.tsv -a2 test_anno_prediction1.tsv -s
test_seqlenfile3.tsv -o output.tsv -c -b -A --quiet
>cat output.tsv
Nseq    …  TP      FP      FN      …  TPR     PPV     SPC     …  SiteTPR SitePPV …
2       …  0.0250  0.1000  0.1000  …  0.2000  0.2000  0.8857  …  0.5000  0.5000  …
>python slalom.py -a1 test_anno_benchmark.tsv -a2 test_anno_prediction2.tsv -s
test_seqlenfile3.tsv -o output.tsv -c -b -A --quiet
>cat output.tsv
Nseq    …  TP      FP      FN      …  TPR     PPV     SPC     …  SiteTPR SitePPV …
2       …  0.1000  0.0250  0.0250  …  0.8000  0.8000  0.9714  …  0.5000  0.5000  …
```

Note that this adjustment may have a quite significant influence on performance metrics, if the sequence lengths are very diverse. However, it affects only the symbol-wise measures and not the site-wise ones. Note also that the adjustment for sequence length differs from sequence-wise averaging:

```
>python slalom.py -a1 test_anno_benchmark.tsv -a2 test_anno_prediction1.tsv -s
test_seqlenfile3.tsv -o output.tsv -c -b -a sequence --quiet
>cat output.tsv
Nseq    …  TP      FP      FN      …  TPR     PPV     SPC     …  SiteTPR SitePPV …
2       …  0.0400  0.0400  0.0400  …  0.5000  0.5000  0.9737  …  0.5000  0.5000  …
```

10. Resolving Overlaps within the Annotations

In some annotation, the sites overlap within one annotation, for example:

```
>cat test_anno1.tsv
seqA  6     10
seqA  8     12
seqA  9     18
seqB  15    25
>cat test_anno2.tsv
seqA  5     11
seqA  7     10
seqA  9     15
seqB  15    19
seqB  21    25
```

By default, all the sites are treated separately, even if they are overlapping or duplicating each other. Therefore, for example, in the first/benchmark annotation of the sequence `seqA`, if counted gross, there are 3 sites with the total length 20 symbols:

```
>python slalom.py -a1 test_anno1.tsv -a2 test_anno2.tsv -s test_seqlenfile1.tsv -o output.tsv -sum -b -E gross -sg --quiet
>cat output.tsv
…
#    SiteNB: Number of sites in the benchmark annotation
#    SiteNP: Number of sites in the prediction annotation
#    SiteNBm: Number of matched sites in the benchmark annotation
#    SiteNPm: Number of matched sites in the prediction annotation
#    SiteLB: Total length of sites in the benchmark annotation
#    SiteLP: Total length of sites in the prediction annotation
…
Seq.    Nseq    … TPR     … SiteNB  SiteNP  SiteNBm SiteNPm SiteLB  SiteLP  SiteTPR …
seqA    1       … 0.8500  … 3       3       3       3       20      18      1.0000  …
seqB    1       … 0.9091  … 1       2       1       2       11      10      1.0000  …
#-------------- … -------- … ------------------------------------------------------- …
Average 1.0000  … 0.8795  … 2.0000  2.5000  2.0000  2.5000  15.500  14.000  1.0000  …
Sum     2       …         … 4       5       4       5       31      28              …
```

If this is not desired behavior, the overlaps can be resolved. Resolving is performed for each annotation individually and is regulated through the options `-a1r`/`--anno1file_resolve` and `-a2r`/`--anno2file_resolve` respectively. There are three alternatives to the default behavior.

First, all the overlapping sites can be merged prior to further calculations. For this, the value `merge` should be passed. The merging has the same affect on symbol-wise statistics as counting symbol-resolved. However, unlike the symbol-resolved mode, the explicit merging affects also the site-wise statistics (for sake of calculating symbol fractions, the displayed total site length is also affected by the operating mode; however, there is no impact on site-wise statistics, like TPR or PPV). Consecutively, there is only one site `6`-`18` of length 13 upon merging in the first annotation of `seqA`:

```
>python slalom.py -a1 test_anno1.tsv -a2 test_anno2.tsv -s test_seqlenfile1.tsv -o output.tsv -sum -c -b -sg --quiet
>cat output.tsv
Seq.    Nseq    … TPR     … SiteNB  SiteNP  SiteNBm SiteNPm SiteLB  SiteLP  SiteTPR …
seqA    1       … 0.7692  … 3       3       3       3       13      11      1.0000  …
seqB    1       … 0.9091  … 1       2       1       2       11      10      1.0000  …
Average 1.0000  … 0.8392  … 2.0000  2.5000  2.0000  2.5000  12.000  10.500  1.0000  …
Sum     2       …         … 4       5       4       5       24      21              …
>python slalom.py -a1 test_anno1.tsv -a2 test_anno2.tsv -s test_seqlenfile1.tsv -o output.tsv -sum -c -b -sg -a1r merge -a2r merge --quiet
>cat output.tsv
Seq.    Nseq    … TPR     … SiteNB  SiteNP  SiteNBm SiteNPm SiteLB  SiteLP  SiteTPR …
seqA    1       … 0.7692  … 1       1       1       1       13      11      1.0000  …
seqB    1       … 0.9091  … 1       2       1       2       11      10      1.0000  …
Average 1.0000  … 0.8392  … 1.0000  1.5000  1.0000  1.5000  12.000  10.500  1.0000  …
Sum     2       …         … 2       3       2       3       24      21              …
```

Second, from each overlapping group, only the last site can be retained, while the others are discarded. For this, the value `last` should be passed. In this case, there is only the site `9`-`18` of length 10 is left:

```
>python slalom.py -a1 test_anno1.tsv -a2 test_anno2.tsv -s test_seqlenfile1.tsv -o output.tsv -sum -c -b -sg -a1r last -a2r last --quiet
>cat output.tsv
Seq.    Nseq    … TPR     … SiteNB  SiteNP  SiteNBm SiteNPm SiteLB  SiteLP  SiteTPR …
seqA    1       … 0.7000  … 1       1       1       1       10      7       1.0000  …
seqB    1       … 0.9091  … 1       2       1       2       11      10      1.0000  …
Average 1.0000  … 0.8045  … 1.0000  1.5000  1.0000  1.5000  10.500  8.5000  1.0000  …
Sum     2       …         … 2       3       2       3       21      17              …
```

Finally, only the first sites of each overlapping group can be retained, by providing the value `first`. This time, only the site `6`-`10` of length 5 is retained:

```
>python slalom.py -a1 test_anno1.tsv -a2 test_anno2.tsv -s test_seqlenfile1.tsv -o
output.tsv -sum -c -b -sg -a1r first -a2r first --quiet
>cat output.tsv
Seq.    Nseq   … TPR    … SiteNB SiteNP SiteNBm SiteNPm SiteLB SiteLP SiteTPR …
seqA    1      … 1.0000 … 1      1      1       1       5      7      1.0000  …
seqB    1      … 0.9091 … 1      2      1       2       11     10     1.0000  …
Average 1.0000 … 0.9545 … 1.0000 1.5000 1.0000  1.5000  8.0000 8.5000 1.0000  …
Sum     2      …        … 2      3      2       3       16     17             …
```

Note that there are no overlapping sites in the sequence `seqB`. Consecutively, its metrics are affected neither by overlap resolving nor by mode switching. Note also that after any of three kinds of resolving there are no overlapping sites left. Therefore, both the symbol-resolved and gross modes will produce equivalent results upon resolving.

11. Overlap Criteria between the Annotations

Overlap criteria are of crucial importance for calculating site-wise metrics. By default, any overlap – even a single symbol – is sufficient to count a pair of sites from different annotations as matched. This is reason, why the site-wise TPR always remained 1.0 in the previous section: regardless of which sites were retained, there was always an overlap. However, often a more sophisticated approach is needed.

Consider the following two annotations:

```
>cat test_anno5.tsv
2      6
20     22
37     47
>cat test_anno6.tsv
3      7
16     30
37     41
43     48
```

The site `2`-`6` from the first/benchmark annotation corresponds to the site `3`-`7` from the second/prediction annotation fairly good: the overlap is 80% (but which still may be not good enough depending on the context). The site `20`-`22` is completely covered; however, the partner `16`-`30` is much longer: 3 symbols vs. 15. Such situations are typical for weak predictors, which are trying to capture the benchmark sites just by chance. Such a match may be considered good in some context but not in the other. The site `37`-`47` is covered on 10 symbols out of 11; however, the coverage is in two patches of length 5 each. Again, it is not possible to say, if this site is recalled without knowing the context and the specific question asked about the data.

SLALOM offers considerable flexibility while considering site matching criteria by allowing to set several types of constraints on overlap of putatively matched sites.

The easiest constraint is the minimal number of symbols in the overlap. The desired value should be passed with the option `-Os`/`--overlap_symbols`. In this example, at least 4 symbols are required:

```
>python slalom.py -a1 test_anno5.tsv -a2 test_anno6.tsv -l 50 --single_sequence -o output.tsv -b -Os 4 --quiet
>cat output.tsv
```
…
```
#     SiteNB: Number of sites in the benchmark annotation
#     SiteNP: Number of sites in the prediction annotation
#     SiteNBm: Number of matched sites in the benchmark annotation
#     SiteNPm: Number of matched sites in the prediction annotation
```
…

Nseq	…	TPR	PPV	…	SiteNB	SiteNP	SiteNBm	SiteNPm	…	SiteTPR	SitePPV	…
1	…	0.8947	0.5484	…	3	4	2	3	…	0.6667	0.7500	…

For the benchmark, the sites `2`-`6` and `37`-`47` are matched but not the site `20`-`22`: although it is completely covered, it is only 3 symbols long and therefore can never match with the constraint of at least 4 symbols in overlap. This leads to the site-wise recall of 2/3. For the prediction, all the sites except for `16`-`30` got their overlap of at least 4 symbols. This leads to the site-wise precision of 3/4.

Another type of constraint is the minimal part of a site to overlap. The desired value should be passed with the option `-Op`/`--overlap_part` as a fraction (not percentage). In this example, at least 80% overlap is required:

```
>python slalom.py -a1 test_anno5.tsv -a2 test_anno6.tsv -l 50 --single_sequence -o output.tsv -b -Op 0.8 -c --quiet
>cat output.tsv
```

Nseq	…	TPR	PPV	…	SiteNB	SiteNP	SiteNBm	SiteNPm	…	SiteTPR	SitePPV	…
1	…	0.8947	0.5484	…	3	4	3	4	…	1.0000	1.0000	…

This time, the prediction is reported site-wise perfect. This is because by default SLALOM calculates overlapping parts from the shortest of the two sites. Thus, the sites `20`-`22` and `16`-`30` score 100% overlap, although it would be significantly less of the length of the latter. This is the most permissive approach of applying the overlapping constraints.

To change the way, how the constraints are applied, the user should provide the option `-Oa`/`--overlap_apply` with the corresponding value. There are three alternative values to `shortest`.

The most rigorous approach is applying to the longer site of the two, by providing the value `longest`:

```
>python slalom.py -a1 test_anno5.tsv -a2 test_anno6.tsv -l 50 --single_sequence -o output.tsv -b -Op 0.8 -Oa longest -c --quiet
>cat output.tsv
```

Nseq	…	TPR	PPV	…	SiteNB	SiteNP	SiteNBm	SiteNPm	…	SiteTPR	SitePPV	…
1	…	0.8947	0.5484	…	3	4	1	1	…	0.3333	0.2500	…

This time, only the sites `2`-`6` and `3`-`7` could match. The site `20`-`22` was not considered, because the overlap part was calculated by the longer partner `16`-`30`: it is now only 3/15, or 20%.

When applying to either the shorter or the longer site, matches are always symmetric. However, it is not the case, when applying to the site of the annotation currently considered, by providing the value `current`:

```
>python slalom.py -a1 test_anno5.tsv -a2 test_anno6.tsv -l 50 --single_sequence -o output.tsv -b -Op 0.8 -Oa current -c --quiet
>cat output.tsv
```

Nseq	…	TPR	PPV	…	SiteNB	SiteNP	SiteNBm	SiteNPm	…	SiteTPR	SitePPV	…
1	…	0.8947	0.5484	…	3	4	2	3	…	0.6667	0.7500	…

Now, two of the sites from the benchmark annotation got their match: 20-22 was considered but 37-47 still not. This is because the latter may be covered to 80% but not with a single site. For each of the sites 37-41 and 43-48 the overlap is only 5 symbols, or 45.45%. For the prediction, however, the situation was the opposite: no match registered in the pair 20-22 and 16-30 but instead both 37-41 and 43-48 passed the 80% threshold. Note that although the recall and precision have the same value as in the example with the requirement of 4 symbols, the contributing sites are now different.

Finally, if patched coverage – the coverage of one site by many – is also good, the value patched should be provided:

```
>python slalom.py -a1 test_anno5.tsv -a2 test_anno6.tsv -l 50 --single_sequence -o
output.tsv -b -Op 0.8 -Oa patched -c --quiet
>cat output.tsv
Nseq      …  TPR     PPV     …  SiteNB  SiteNP  SiteNBm SiteNPm …  SiteTPR SitePPV …
1         …  0.8947  0.5484  …  3       4       3       3       …  1.0000  0.7500  …
```

This time, the site 37-47 was also matched by satisfying the 80% threshold with the total coverage of 10 symbols from two sites contributing 5 symbols each.

In addition to setting constraints on overlap length, one may also consider the order of sites in a putative pair. Sometimes, additional information about the relation between sites in the annotations is available, which makes only those site pairs meaningful, in which the predicted site appears earlier or later than the benchmark one. To impose such constraints, the user can use the option -On/--overlap_nature with the value either leading or lagging. The option is compatible only with the benchmarking mode; a leading prediction can start no later than the benchmark, and vice versa for the lagging one:

```
>python slalom.py -a1 test_anno5.tsv -a2 test_anno6.tsv -l 50 --single_sequence -o
output.tsv -b -On leading -c --quiet
>cat output.tsv
Nseq      …  TPR     PPV     …  SiteNB  SiteNP  SiteNBm SiteNPm …  SiteTPR SitePPV …
1         …  0.8947  0.5484  …  3       4       2       2       …  0.6667  0.5000  …
>python slalom.py -a1 test_anno5.tsv -a2 test_anno6.tsv -l 50 --single_sequence -o
output.tsv -b -On lagging -c --quiet
>cat output.tsv
Nseq      …  TPR     PPV     …  SiteNB  SiteNP  SiteNBm SiteNPm …  SiteTPR SitePPV …
1         …  0.8947  0.5484  …  3       4       2       3       …  0.6667  0.7500  …
```

If the prediction is leading, the sites 2-6 and 3-7 can no longer match, even if they overlap sufficiently, because the latter begins later than the former. Similarly, if the prediction is declared lagging, the sites 20-22 and 16-30, as well as 37-47 and 43-48 cannot match, because the predicted site begins earlier.

The three constraints can be applied simultaneously; the AND logic will be used.

Note that the three discussed types of constraints do affect only the site-wise metrics and do not influence residue-wise ones.

12. Detailed, Site-wise Statistics as Output

In previous sections of this manual, it was demonstrated that the main output file contains statistics at the group or whole dataset levels. However, in some situations, information on each annotated site is desirable. This may be useful for two purposes. First, the user may wand to understand, how performance metrics on the group and dataset levels arise or why the performance is worse/better

than expected. Second, some sort of mapping between sites in different annotations may be required. Mapping is discussed in more detail in the next section.

SLALOM can provide site-wise statistics in two different output files.

The first file is called 'detailed output'. It is a plain text file and represents a log of SLALOM operations, as it works through the input annotations. The filename should be provided with the option `-od`/`--outfile_detailed`. Using an example from the previous section:

```
>python slalom.py -a1 test_anno5.tsv -a2 test_anno6.tsv -l 50 --single_sequence -o
output.tsv -od output_detailed.txt -b -Op 0.8 -Oa current -c --quiet
>cat output_detailed.txt
Information on the unnamed sequence (length 50 symbols):
    17 symbols are present in both annotations (true positives)
    2 symbols are present exclusively in the benchmark (false negatives)
    14 symbols are present exclusively in the prediction (false positives)
    17 symbols are absent in both annotations (true negatives)
    Site 2-6 of the benchmark: overlaps with site 3-7 of the prediction by 4 symbols
(80% and 80% of the site lengths respectively)
    Site 20-22 of the benchmark: overlaps with site 16-30 of the prediction by 3
symbols (100% and 20% of the site lengths respectively)
    Site 37-47 of the benchmark: no sufficient overlap found
    Site 3-7 of the prediction: overlaps with site 2-6 of the benchmark by 4 symbols
(80% and 80% of the site lengths respectively)
    Site 16-30 of the prediction: no sufficient overlap found
    Site 37-41 of the prediction: overlaps with site 37-47 of the benchmark by 5
symbols (100% and 45% of the site lengths respectively)
    Site 43-48 of the prediction: overlaps with site 37-47 of the benchmark by 5
symbols (83% and 45% of the site lengths respectively)
    There are 3 sites in the benchmark with total length 19 unique symbols
    2 benchmark sites are matched in the benchmark
    1 benchmark site has no match in the benchmark
    There are 4 sites in the prediction with total length 31 unique symbols
    3 prediction sites are matched in the benchmark
    1 prediction site has no match in the benchmark
```

The second file contains essentially the same information, but in a tabular form (the TSV format). It lists all the sites, one site per row. The column names are the following: `Sequence` (SID; empty, if single sequence without an SID is processed), `Annotation` (`b` for the benchmark, `p` for the prediction; in the equal mode: `1` and `2`), `Site begin`, `Site end`, `Overlapped symbols`, `Overlapped perc.`, `Partner overlapped perc.`, `Partner begin`, `Partner end`. They comprise the header row. The filename should be provided with the option `-os`/`--outfile_sites`:

```
>python slalom.py -a1 test_anno5.tsv -a2 test_anno6.tsv -l 50 --single_sequence -o
output.tsv -os output_sitewise.tsv -b -Op 0.8 -Oa current -c --quiet
>cat output_sitewise.tsv
…
        b       2       6       4       80      80      3       7
        b       20      22      3       100     20      16      30
        b       37      47      0       0       0       -       -
        p       3       7       4       80      80      2       6
        p       16      30      0       0       0       -       -
        p       37      41      5       100     45      37      47
        p       43      48      5       83      45      37      47
```

13. Mapping of Site Names/Identifiers between the Annotations

Sometimes, the input annotations contain the same sites (for example, genes in a certain organism), which, however, are derived from different sources with each source providing its own version of the site names (a.k.a. identifiers or accessions). Matching the names in such a situation is known as mapping. As the sites do not necessarily match perfectly between the annotations, the mapping is often ambiguous. Nevertheless, a reasonably good mapping can be produced, if the data quality is good enough and overlapping criteria are set wisely.

Consider the following annotations (assuming both sequences have the length 150):

```
>cat test_anno7.tsv
seqA  21    40    geneA
seqA  61    85    geneB
seqA  101   130   geneC
seqB  16    30    geneD
>cat test_anno8.tsv
seqA  21    40    gene1
seqA  61    88    gene2
seqA  121   150   gene3
```

In them, `geneA` and `gene1` match perfectly, `geneB` and `gene2` match reasonably, `geneC` and `gene3` overlap only modestly, while `geneD` cannot be matched in the second annotation at all. To read in also the site names and consequently show them in the site-wise output, the user should provide the option `-n`/`--site_names`. The overlap quality criterion can be, for example, at least 90% of the current gene:

```
>python slalom.py -a1 test_anno7.tsv -a2 test_anno8.tsv -l 150 -o output.tsv -os
output_mapping.tsv -n -Op 0.9 -Oa current -c --quiet
>cat output_mapping.tsv
```

...

```
seqA    1       21      40      geneA   20      100     100     21      40      gene1
seqA    1       61      85      geneB   25      100     89      61      88      gene2
seqA    1       101     130     geneC   0       0       0       -       -
seqA    2       21      40      gene1   20      100     100     21      40      geneA
seqA    2       61      88      gene2   0       0       0       -       -
seqA    2       121     150     gene3   0       0       0       -       -
seqB    1       16      30      geneD   0       0       0       -       -
```

In comparison to the case, when no site names are provided, the table has two additional columns: column 5 (`Site name`) and column 11 (`Partner name`). By default, the mapping lists all the sites from the input annotations. For sites without a match, the partner name is empty. Nevertheless, the user can limit the output by setting the option `-osd`/`--outfile_sites_diff` to either `matched`, `unmatched` or `discrepant` (the latter will list all but perfect matches, regardless of the overlapping criteria set):

```
>python slalom.py -a1 test_anno7.tsv -a2 test_anno8.tsv -l 150 -o output.tsv -os
output_mapping.tsv -osd matched -n -Op 0.9 -Oa current -c --quiet
>cat output_mapping.tsv
```

...

```
seqA    1       21      40      geneA   20      100     100     21      40      gene1
seqA    1       61      85      geneB   25      100     89      61      88      gene2
seqA    2       21      40      gene1   20      100     100     21      40      geneA
>python slalom.py -a1 test_anno7.tsv -a2 test_anno8.tsv -l 150 -o output.tsv -os
output_mapping.tsv -osd unmatched -n -Op 0.9 -Oa current -c --quiet
>cat output_mapping.tsv
```

...

```
seqA    1       101     130     geneC   0       0       0       -       -
seqA    2       61      88      gene2   0       0       0       -       -
seqA    2       121     150     gene3   0       0       0       -       -
seqB    1       16      30      geneD   0       0       0       -       -
```

```
>python slalom.py -a1 test_anno7.tsv -a2 test_anno8.tsv -l 150 -o output.tsv -os
output_mapping.tsv -osd discrepant -n -Op 0.9 -Oa current -c --quiet
>cat output_mapping.tsv
```
...
```
seqA    1    61     85     geneB    25    100    89    61    88    gene2
seqA    1    101    130    geneC    0     0      0     -     -
seqA    2    61     88     gene2    0     0      0     -     -
seqA    2    121    150    gene3    0     0      0     -     -
seqB    1    16     30     geneD    0     0      0     -     -
```

Discrepant and unmatched sites are important, if one wants to concentrate on differences: for example, when comparing two versions of the same genome annotation.

Note that in the simplified modes (GenBank or BED) the decision on reading the site name is done automatically on the basis of the structure of the input files.

SLALOM can also perform mapping of elements that do not overlap at all. This becomes useful, for example, if one wants to associate genes with other genomic elements, like, e.g., promoters. If these are known to precede the associated genes locating to a window of certain length before a gene start position, the start positions can be imaginary 'shifted' in order to register an 'overlap'. The desirable amount of symbols should be provided with the respective option `-a1bs`/`--anno1file_begin_shift` or `-a2bs`/`--anno2file_begin_shift`. As the shift in the direction of the sequence start is needed, a negative value should be provided:

```
>cat test_anno9.tsv
seqA  16    19    elemA
seqA  53    57    elemB
seqA  95    98    elemC
seqB  7     12    elemD
seqB  61    64    elemE
>python slalom.py -a1 test_anno7.tsv -a2 test_anno9.tsv -l 150 -o output.tsv -os
output_mapping.tsv -n -a1bs -10 -c --quiet
>cat output_mapping.tsv
```
...
```
seqA    1    11    40     geneA    4    13     100    16    19     elemA
seqA    1    51    85     geneB    5    14     100    53    57     elemB
seqA    1    91    130    geneC    4    10     100    95    98     elemC
seqA    2    16    19     elemA    4    100    13     11    40     geneA
seqA    2    53    57     elemB    5    100    14     51    85     geneB
seqA    2    95    98     elemC    4    100    10     91    130    geneC
seqB    1    6     30     geneD    6    24     100    7     12     elemD
seqB    2    7     12     elemD    6    100    24     6     30     geneD
seqB    2    61    64     elemE    0    0      0      -     -
```

The same effect can be also reached by extending the elements in the second annotation through the similar option `-a2es`/`--anno2file_end_shift`.

In this case, the outputted overlapping percentages do not make much sense any longer, as the 'gene length' was artificially changed. However, the shift options can be also used to correct the input: for example, if symbol counting starts from 0 instead from 1, the value `1` should be passed to adjusting the sites to the SLALOM conventions. Note that 1 is automatically added to the read start positions in the BED simplified mode; independently of the provided shift values.

14. Circular Sequences as Input

In some areas of science, like, e.g., bacterial genomics, circular sequences are very common.

A usual approach to annotate such a sequence is to introduce an imaginary cut at some position, thus converting it to a linear sequence. Working with a circular sequence is therefore the same, as long as the cut does not go through an annotated site. If that happens, however, the cut-through site must be still represented as a single entity. To achieve this, one normally marks the end position with a symbol numbers that are greater than the sequence length. Alternatively, one can a use non-positive start symbol number.

Let us consider the following annotations of one site each in a sequence of length 50. The last annotations actually refer to the same site but with different notation:

```
>cat test_anno_circular1.tsv
1     10
>cat test_anno_circular2.tsv
48    59
>cat test_anno_circular3.tsv
-2    9
```

By default, SLALOM considers input sequences linear and raises an error, when a symbol number out of scope of the sequence is detected:

```
>python slalom.py -a1 test_anno_circular1.tsv -a2 test_anno_circular2.tsv -l 50 --single_sequence -o output.tsv -b -c --quiet
Error: Error while parsing the line 1 of the file "test_anno_circular2.tsv". Site end position cannot exceed the sequence length
```

To treat the input as circular, the user should provide the value `circular` with the option `-e`/`--end_overflow_policy`:

```
>python slalom.py -a1 test_anno_circular1.tsv -a2 test_anno_circular2.tsv -l 50 --single_sequence -o output.tsv -od output_detailed.txt -e circular -b -c --quiet
>cat output.tsv
Nseq     …  TPR      PPV      …  SiteNB   SiteNP   SiteNBm  SiteNPm  SiteLB   SiteLP   …
1        …  0.9000   0.7500   …  1        1        1        1        10       12       …
>cat output_detailed.txt
…
    Site 1-10 of the benchmark: overlaps with site 48-59 of the prediction by 9 symbols (90% and 75% of the site lengths respectively)
    Site 48-59 of the prediction: overlaps with site 1-10 of the benchmark by 9 symbols (75% and 90% of the site lengths respectively)
…
>python slalom.py -a1 test_anno_circular1.tsv -a2 test_anno_circular3.tsv -l 50 --single_sequence -o output.tsv -e circular -b -c --quiet
>cat output.tsv
Nseq     …  TPR      PPV      …  SiteNB   SiteNP   SiteNBm  SiteNPm  SiteLB   SiteLP   …
1        …  0.9000   0.7500   …  1        1        1        1        10       12       …
```

The overlap of the two sites and performance metrics are calculated as usual, as if there were no cut through the second one.

15. Custom Tabular Files as Input

In the earlier sections of this manual, it was shown that SLALOM can process input files in some predefined formats without further specifications, as well as tabular files, which satisfy certain conditions. These conditions are the following:

- The column delimiter is tab
- The columns are following the order SID - GID - start - end - name (non-specified columns omitted) for the annotation files, SID - length for the sequence length table, and SID - GID for the group mapping file
- There are no further columns before or between the specified ones (additional columns to the right will be ignored)
- There are no header rows
- Quotes are not part of the identifiers and names (although tabs may be if quoted)

Therefore, the user always has an option to convert the input files in one of the supported formats to process them with SLALOM in an easy way. However, this is not always convenient, as there are plenty of tabular data files – available for download or outputted by software tools – that do not strictly conform to requirements of the supported format. Furthermore, the user may encounter situations, when the annotations come from different sources and therefore have different formats. To also offer flexibility for these cases, SLALOM has extended options allowing it to read virtually any kind of tabular data files.

The format options are combinations of prefixes and suffixes. The prefixes correspond to the file name options: `-a1` for the first/benchmark annotation, etc. The suffixes are given in the following table:

Short version	Long version	Description
`d`	`delim`	Specifies the column delimiter (the following delimiters are supported: tab, space, comma, dot, semicolon, colon, slash). Consecutive delimiters mark empty values, unless empty string is passed as delimiter. In that case, space is used but with leading and trailing spaces removed and consecutive spaces collapsed
`c`	`colnumbers`	Specifies the column numbers in the following order: start - end - SID - GID - name for the annotation files; SID - length (SID - start - end for time series) for the sequence length table; SID - GID for the group mapping
`h`	`headers`	Specifies the number of header rows at the beginning of the file
`q`	`quotes`	Triggers the literal treatment of quotes (quoted delimiters are treated as delimiters; single – but not double – quotes can be part of identifiers and names)

A prefix is combined with a suffix to form one of the 16 possible options. For the long options, the extra underscore is added as separator. Therefore, to specify, for example, that the second annotation file has three header rows that need to be skipped, the user should provide the value `3` with the option `-a2h`/`--anno2file_headers` (e.g., `-a2h 3`). The column numbers are integers, so that the leftmost column has the number 1. They must be provided comma-separated without additional spaces. For example, if the sequence lengths are read from a file that contains the identifiers in the fifth column and the length values in the third column, the user should provide the value `5,3` with the option `-sc`/`--seqlenfile_colnumbers` (e.g., `-sc 5,3`).

This approach allows that same files and/or same columns may be used several times if the input format requires so. In the following example, the sequence lengths and the group mapping are read from the annotation files:

```
>cat test_format1.txt
Number;ID;Quality;Score;Start;End
1;ABC;Good;0.76;17;55
2;ABC;Good;1.02;58;70
3;DEF;Good;1.21;12;28
4;XYZ;Bad;0.18;101;108
>cat test_format2.csv
#This file was generated by …
Origin,Sequence,Length,Peak Value,Average value,Start,End
Natural,ABC,100,8.4,2.0,48,62
Artif.,DEF,120,9.1,1.9,26,40
Artif.,DEF,120,6.2,0.9,69,85
Artif.,XYZ,150,11.5,3.1,108,122
>python slalom.py -a1 test_format1.txt -a1h 1 -a1c 5,6,2 -a1d ";" -a2 test_format2.csv
-a2h 2 -a2c 6,7,2 -a2d "," -s test_format2.csv -sh 2 -sc 2,3 -sd "," -m
test_format1.txt -mh 1 -mc 2,3 -md ";" -o output.tsv -nOg -c --quiet
>cat output.tsv
Group   Nseq    P1      P2      …  SiteN1  SiteN2  SiteN1m SiteN2m SiteL1  SiteL2  …
Good    2       0.3136  0.2136  …  3       3       3       2       69      47      …
Bad     1       0.0533  0.1000  …  1       1       1       1       8       15      …
Average 1.5000  0.1835  0.1568  …  2.0000  2.0000  2.0000  1.5000  38.500  31.000  …
```

For the first calculation, the column `Quality` of the file `test_format1.txt` was used for grouping the sequences. Therefore, the file was provided twice: as the first annotation (`-a1 test_format1.txt`) and as the group mapping (`-m test_format1.txt`); the file `test_format2.csv` was also provided twice: as the second annotation (`-a2 test_format2.csv`) and as the sequence length table (`-s test_format2.csv`). The column `Quality` in the former file has number 3, while the SiDs are in the column `ID` with the number 2, which results in `-mc 2,3`. Providing the option `-nOg`/`--non_overlapping_groups` allows for omitting the GIDs in the annotation files (in fact, it would be not possible to specify these GIDs in the second annotation file, as the quality information is missing there). Therefore, only three columns are read from each of the annotations: start position, end position, and SID. In the file `test_format1.txt`, the start positions are in column number 5, the end positions are in column number 6, and the SIDs are in column number 2, which results in `-a1c 5,6,2`. Following the same logic, the option for the second annotation is `-a2c 6,7,2`. The sequence length values are provided in the same file in column number 3, which results in `-sc 2,3`.

The next two calculations are conducted in a similar way:

```
>python slalom.py -a1 test_format1.txt -a1h 1 -a1c 5,6,2 -a1d ";" -a2 test_format2.csv
-a2h 2 -a2c 6,7,2 -a2d "," -s test_format2.csv -sh 2 -sc 2,3 -sd "," -m
test_format2.csv -mh 2 -mc 2,1 -md "," -o output.tsv -nOg -c --quiet
>cat output.tsv
Group   Nseq    P1      P2      …  SiteN1  SiteN2  SiteN1m SiteN2m SiteL1  SiteL2  …
Natural 1       0.5200  0.1500  …  2       1       2       1       52      15      …
Artif.  2       0.0926  0.1741  …  2       3       2       2       25      47      …
Average 1.5000  0.3063  0.1620  …  2.0000  2.0000  2.0000  1.5000  38.500  31.000  …
>python slalom.py -a1 test_format1.txt -a1h 1 -a1c 5,6,2 -a1d ";" -a2 test_format2.csv
-a2h 2 -a2c 6,7,2 -a2d "," -s test_format2.csv -sh 2 -sc 2,3 -sd "," -o output.tsv --
sequences_as_groups -c --quiet
>cat output.tsv
Seq.    Nseq    P1      P2      …  SiteN1  SiteN2  SiteN1m SiteN2m SiteL1  SiteL2  …
ABC     1       0.5200  0.1500  …  2       1       2       1       52      15      …
DEF     1       0.1417  0.2667  …  1       2       1       1       17      32      …
XYZ     1       0.0533  0.1000  …  1       1       1       1       8       15      …
Average 1.0000  0.2383  0.1722  …  1.3333  1.3333  1.3333  1.0000  25.667  20.667  …
```

16. Time Series as Input

SLALOM is a generic statistical tool. Although it was originally developed to work with SCEs in protein and DNA sequences, its abstract design allows applying it to virtually any kind of sequences, in biology and beyond.

Working with time series follows the same logic as analyzing the ranges in alphabetical sequences. Indeed, time intervals can be viewed as SCEs in the sequences of time units. For processing time series, SLALOM is started with providing the option `-t`/`--time_unit`. The four following time units with the corresponding values are supported: second (`sec`), minute (`min`), hour (`hour`), and day (`day`). Now, the start and end positions in the annotation files are replaced with time stamps. For the sequence length table, the length values are replaced with pairs of sequence start and end time stamps (so that the sequence length table now contains three columns instead of two). The following two date formats are supported: `dd.mm.YYYY` and `mm/dd/YYYY`. The optional time is expected after the space in the format `HH:MM` or `HH:MM:SS`; AM/PM classification is currently not supported. If the time is not specified, `00:00:00` is assumed.

After the internal conversion of time intervals to symbols, the workflow and the output are the same as for a general SLALOM case. Time sequences as well as individual intervals can also overlap and the usual rules for overlap resolving will be applied:

```
>cat test_time_lines.tsv
PlaceA  02/01/2015 22:45        02/02/2015 09:15
PlaceB  02/02/2015 02:10        02/03/2015 00:45
PlaceC  02/03/2015 17:05        02/03/2015 22:10
>cat test_anno_events1.tsv
PlaceA  02/01/2015 23:05        02/01/2015 23:30
PlaceA  02/02/2015 03:10        02/02/2015 04:00
PlaceB  02/02/2015 03:15        02/02/2015 04:05
>cat test_anno_events2.tsv
PlaceA  02/01/2015 23:10        02/01/2015 23:40
PlaceB  02/02/2015 03:15        02/02/2015 04:10
PlaceB  02/02/2015 03:45        02/02/2015 05:00
PlaceC  02/03/2015 18:35        02/03/2015 19:10
>python slalom.py -t min -s test_time_lines.tsv -a1 test_anno_events1.tsv -a2
test_anno_events2.tsv -o output.tsv --sequences_as_groups -sum -c --quiet
>cat output.tsv
Seq.    Nseq    P1      P2      …  SiteN1  SiteN2  SiteN1m SiteN2m SiteL1  SiteL2  …
PlaceA  1       0.1190  0.0476  …  2       1       1       1       75      30      …
PlaceB  1       0.0369  0.0775  …  1       2       1       2       50      105     …
PlaceC  1       0.0000  0.1148  …  0       1       0       0       0       35      …
Average 1.0000  0.0520  0.0800  …  1.0000  1.3333  0.6667  1.0000  41.667  56.667  …
Sum     3                       …  3       4       2       3       125     170     …
```

Sequence grouping and all the previously discussed associated option work also for time series.

17. Summary of Available Command Line Options for SLALOM

Type	Default	Description
**Main input/output files**		
`-s, --seqlenfile`		
string	(empty)	Sequence length table file. Maps SIDs to corresponding sequence lengths (or, in case of time series, start and finish points). Must be in

		tabular format. The file shall not be provided, if the length of all sequences is specified elsewhere
`-m, --mapfile`		
string	(empty)	Mapping file. Maps GIDs and SIDs. Must be in tabular format
`-a1, --anno1file`		
string	(required)	First/benchmark annotation file. Lists all sites from the first annotation, one site per line. Must be in tabular format, unless `--genbank` is specified
`-a2, --anno2file`		
string	(required)	Second (prediction) annotation file. See the option `-a1` for details
`-o, --outfile`		
string	(required)	Output TSV file with calculated performance measures, for each group and dataset-wide
**Simplified modes**		
`--genbank`		
bool	`false`	Read the annotations in GenBank format. Sequence length will be read automatically
`--bed`		
bool	`false`	Read the annotations in BED format
**Operation mode setup**		
`-b, --benchmarking`		
bool	`false`	Activate the benchmarking mode, i.e., treat the first annotation as benchmark and the second as prediction (default: treat the annotations symmetrically)
`-E, --enrichment_count`		
integer or string	0	Switch between symbol-resolved, gross, and enrichment modes. Must be a non-negative integer or `gross`. If 0, activates the symbol-resolved mode. If >=1, activates the enrichment mode with minimal number of occurrences of a symbol position to count it as enriched. If `gross`, activates the gross mode
**Core algorithm controls**		
`-Os, --overlap_symbols`		
integer	1	Minimal number of symbols required for a site from the annotation currently considered to be overlapping with a site/sites from the other annotation to be counted as a match. Must be a positive integer
`-Op, --overlap_part`		
float	0.0	Minimal fraction of the length of a site from the annotation currently considered required to overlap with site/sites from the other annotation to be counted as a match. Must be in range [0,1]
`-Oa, --overlap_apply`		
enum	`shortest`	The principle to apply the minimal number of symbols and the minimal part:<br>- `shortest`: to the shortest of two sites (one site from the first annotation and the other one from the second)<br>- `longest`: to the longest of two sites<br>- `current`: to the site from the annotation currently considered; sites from another annotation are considered one at a time

		- `patched`: to the sites from the annotation currently considered; allowing multiple sites from the other annotation to contribute simultaneously
`-On, --overlap_nature`		
enum	`neutral`	The required overlap nature of the predicted sites:<br>- `neutral`: count site overlaps regardless of the order of their start positions<br>- `leading`: only count a predicted site as match, if its start position is earlier or the same as that of the respective benchmark site<br>- `lagging`: only count predicted site as match, if the begin position is later or the same as that of the respective benchmark site<br>The default value can be changed only in the benchmarking mode and only for non-circular sequences
`-a, --averaging`		
enum	`group`	Order of averaging when calculating performance measures:<br>- `sequence`: calculate the measures for each sequence individually, then simple-average group-wide, then simple-average dataset-wide (macro-macro averaging)<br>- `group`: sum the counts group-wide, then calculate the measures, then simple-average dataset-wide (micro-macro averaging)<br>- `dataset`: sum the counts dataset-wide and calculate the measures (micro-micro averaging); for results on individual groups, apply macro averaging
`-A, --adjust_for_seqlen`		
bool	`false`	Adjust the counts for each sequence for its length, then average the adjusted values (default: sum the counts). Affects only symbol-wise measures. Not compatible with sequence-wide averaging.
**Input file format**		
`-sd, --seqlenfile_delim`		
char	tab	Column delimiter in the sequence length table. Allowed delimiters: space, tab, comma, dot, colon, semicolon, slash. If an empty string is passed, space will be used, multiple spaces will be collapsed, and leading, as well as trailing spaces will be removed removed
`-md, --mapfile_delim`<br>`-a1d, --anno1file_delim`<br>`-a2d, --anno2file_delim`		
(see the option `-sd`)		
`-sh, --seqlenfile_headers`		
integer	0	Number of header rows to skip in the sequence length table. Must be a non-negative integer
`-mh, --mapfile_headers`<br>`-a1h, --anno1file_headers`<br>`-a2h, --anno2file_headers`		
(see the option `-sh`)		
`-sc, --seqlenfile_colnumbers`		
list of integers	(adjusted)	Comma-delimited list of column numbers (left-most column has

		number 1) in the sequence length table with SIDs and sequence length in that order. In case of time series, start and finish points must be provided instead of the length. The default value is adjusted according to the expected columns
-mc, --mapfile_colnumbers		
list of integers	1,2	Comma-delimited list of column numbers (left-most column has number 1) in the group-mapping file with SIDs and GIDs in that order
-a1c, --anno1file_colnumbers		
list of integers	(adjusted)	Comma-delimited list of column numbers (left-most column has number 1) in the first annotation file with site start positions, site end positions, SIDs, GIDs, and site names in that order (with those not provided skipped). The default value is adjusted according to the expected columns
-a2c, --anno2file_colnumbers		
(see the option -a1c)		
-sq, --seqlenfile_quotes		
bool	false	Treat single quotes (apostrophes) in the sequence length table file literally. By default, delimiters inside quotes (both single and double) are treated as belonging to fields and quotes themselves are ignored. Double quotes can never be part of read input; their presence in the file triggers an error, if this option is true
-mq, --mapfile_quotes<br>-a1q, --anno1file_quotes<br>-a2q, --anno2file_quotes		
(see the option -sq)		
**Alternative input options**		
-l, --seqlen_value		
integer	0	Length of all sequences. Must be a non-negative integer. If 0, this option is ignored. If positive, the sequence length table shall not be provided; the length of every sequence is set to the specified value; all SIDs encountered in the annotations and the group-mapping file will be used to build the sequence length table. With time series options -ts and -tf shall be used instead
-ss, --single_sequence		
bool	false	Process single sequence. SIDs shall not be provided if this option is activated
-ts, --timeseries_start		
string	(empty)	Start of all time series. If empty, this option is ignored. Must be used together with the option -tf. For details, see the option -l
-tf, --timeseries_finish		
string	(empty)	Finish of all time series. For details, see the option -ts
-a1as, --anno1file_all_sequences		
bool	false	Consider all the sites from the first annotation as belonging to all the sequences. SIDs shall not be provided if this option is activated. This option cannot be selected for both annotations simultaneously
-a2as, --anno2file_all_sequences		
(see the option -a1as)		
-a1ag, --anno2file_all_groups		

bool	`false`	Consider all the sites from the first annotation as belonging to sequences with provided SIDs in all the groups. If this option is activated, GIDs shall not be provided, although the group mapping must be provided. This option can be selected for both annotations, but it is not compatible with considering all the sites as belonging to all the sequences
`-a2ag`, `--anno2file_all_groups`		
(see the option `-a1ag`)		
`-sg`, `--sequences_as_groups`		
bool	`false`	Generate respective 1-sequence groups for all the SIDs. If this option is activated, neither the group-mapping file nor GIDs shall be provided
`-nOg`, `--non_overlapping_groups`		
bool	`false`	Define the groups provided in the group-mapping file as non-overlapping. If this option is activated, GIDs in the annotation files shall not be provided
**Input controls**		
`-n`, `--site_names`		
bool	`false`	Also read in site names from the annotation files. This option does not affect calculations, but the names will be shown in the detailed and site-wise output files. The names can be duplicated, but cannot be empty or contain double quotes
`-t`, `--time_unit`		
enum	`none`	Time unit if the sequences are time series (`none` otherwise). Supported values: `sec`, `min`, `hour`, `day`
`-a1r`, `--anno1file_resolve`		
enum	`all`	The rule to resolve site overlaps within the first annotation:<br>- `all`: leave all sites untouched<br>- `first`: retain only the first site from an overlapping group<br>- `last`: retain only the last site from an overlapping group<br>- `merge`: merge an overlapping group to single site<br>If `first` or `last` is selected, only one site will be retained from the whole group, even if the first and last sites of the group do not overlap. For circular sequences, only `all` and `merge` are supported
`-a2r`, `--anno2file_resolve`		
(see the option `-a1r`)		
`-a1bs`, `--anno1file_begin_shift`		
integer	0	Constant shift in symbols of site start positions in the first annotation; this number will be added to all the start positions. If strand detection is activated, this option affects actual end positions in the reverse strand. If frame detection is activated, the frames are not affected by this value
`-a2bs`, `--anno2file_begin_shift`		
(see the option `-a1bs`)		
`-a1es`, `--anno1file_end_shift`		
integer	0	Constant shift in symbols of site end positions in the first annotation. For details, see the option `-a1bs`
`-a2es`, `--anno2file_end_shift`		
(see the option `-a1es`)		

`-e, --end_overflow_policy`		
enum	`forbid`	The rule for overflowing site end positions, which is required if a site start is non-positive or a site end exceeds the sequence length:<br>- `forbid`: terminate program with the error message<br>- `trim`: trim the site to fit the sequence; ignore, if both, begin and end exceed the sequence length<br>- `ignore`: ignore the site completely<br>- `circular`: make overflowing sites reappear at the other end of the sequence; the difference between the end and the begin must still be non-negative and less than the sequence length<br>For time series, `circular` is not supported
`-z, --zero_for_na`		
bool	`false`	Treat `nan` values as zeros to calculate averages (default: exclude from averaging)
`-min, --min_group_size`		
integer	1	Minimal number of sequences of a group. Smaller groups will be ignored. Must be a positive integer
`-max, --max_group_size`		
integer	0	Maximal number of sequences of a group. Larger groups will be ignored. Must be a non-negative integer. If 0, the size is unlimited
`-d, --detect`		
enum	`none`	If `strand`, detect DNA strand based on the information in the file. If `frame`, in addition detect reading frames on the basis of the remainder of division by 3 of the gene start position in the corresponding strand. The frames are not adjusted by user-specified shifts (see the option `-a1bs` for details). The default value can be changed only in the simplified *GenBank* or *BED* modes
**Additional output files**		
`-od, --outfile_detailed`		
string	(empty)	Output file with details at the sequence and group levels
`-os, --outfile_sites`		
string	(empty)	Output file with site-wise statistics including match information
`-ou, --outfile_union`		
string	(empty)	Output TSV file with the union of the two input annotations
`-oi, --outfile_intersection`		
string	(empty)	Output TSV file with the intersection of the two input annotations
`-oc1, --outfile_complement_1`		
string	(empty)	Output TSV file with the complement of the first/benchmark annotation
`-oc2, --outfile_complement_2`		
string	(empty)	Output TSV file with the complement of the second/prediction annotation
`-ore1, --outfile_rel_enrichment_1`		
string	(empty)	Output TSV file with the sites of relative enrichment in the first/benchmark annotation
`-ore2, --outfile_rel_enrichment_2`		
string	(empty)	Output TSV file with the sites of relative enrichment in the second/prediction annotation

Output options		
-osd, --outfile_sites_diff		
enum	all	Limit the site-wise statistics to:<br>- all: do not limit<br>- matched: show only sites that do have a match in the other annotation under the provided criteria<br>- unmatched: show only sites that do not have a match in the other annotation under the provided criteria<br>- discrepant: show only sites that do not have a perfect reciprocal match in the other annotation. Not applicable, if overlap criteria are applied to patched<br>This option applies only in a non-enrichment mode
-c, --clean		
bool	false	Produce cleaned output TSV without the commented lines. This file will contain a single header line with column names and will be ready for import into various data analysis software packages
-sort, --sort_output		
bool	false	Sort the main output file by the group names (default: sort by the order of appearance in the input). Applicable only, if groups are defined
-sum, --calculate_sums		
bool	false	Calculate sums of integer counts in addition to averages. Adds additional row in the main output table. Applicable only, if groups are defined
**Other options**		
-preparse, --preparse_mapfile		
bool	false	Pre-parse the group-mapping file before parsing the sequence length file. This option improves performance, if the number of SIDs in the sequence length file is much larger than in the mapping file
-w, --warning_level		
integer	1	Warning level:<br>- 0: switch off warnings<br>- 1: show all warnings
-q, --quiet		
bool	false	Do not print the progress in the command line
